# Supplementary material for: Integrative analyses reveal signaling pathways underlying familial breast cancer susceptibility
Source: Mol Syst Biol. 2016 Mar 11;12(3):860. doi: 10.15252/msb.20156506 (PMC4812528; doi:10.15252/msb.20156506)

Integrative analyses reveal signaling pathways underlying familial breast cancer susceptibility

Appendix

Tables **2**
Table S1: Data source summary. 2
Table S2: Summary of top-performing pathways in gene-expression and exome-sequencing analyses. 3
Table S3: Relative band area of each protein in the gel quantified by ImageJ software. 4
Figures **5**
Figure S1: Distribution of ages for patients in the Utah cohort. 5
Figure S2: Summary of variant effects on protein function. 6
Figure S3: Summary of DNA alterations that resulted from potentially pathogenic variants. 7
Figure S4: Heatmaps showing median expression levels for Utah and Ontario women who developed FBC and for women who did not, separated according to BRCA1 and BRCA2 mutation status, for two key pathways. 8
Figure S5: Per-sample DNA variants observed in two key pathways. 9

Tables

Table S1: Data source summary. Genomic data were acquired from various sources and profiled using gene-expression microarrays and/or exome-capture sequencing.

A) Gene expression

Description	*Tissue Source*	*Platform*	*# Samples*	*Facility*	*# Genes*
Utah	PBMCs	Affymetrix Exon array	124	Boston U.	25,195
Ontario	PBMCs	Affymetrix Exon array	73	Duke U.	25,195
Lim, et al.	Pre-neoplastic breast	Illumina HumanWG-6 beadchip	20	N/A	25,186
Bellacosa, et al.	Pre-neoplastic breast	Affymetrix HG U133 Plus 2.0	18	N/A	17,833

B) DNA sequencing

Description	*Tissue Source*	*# Samples*
Utah	PBMCs	35

Table S2: Summary of top-performing pathways in gene-expression and exome-sequencing analyses. This table lists pathways that performed best within the Utah and Ontario data sets. Pathways shown here had a rank p-value less than 0.05. See Supplementary Data File 1 for a complete list.

Cell adhesion
- REACTOME INTEGRIN CELL SURFACE INTERACTIONS
- KEGG SMALL CELL LUNG CANCER
- BIOCARTA BCR PATHWAY
- REACTOME CELL SURFACE INTERACTIONS AT THE VASCULAR WALL
- BIOCARTA EDG1 PATHWAY
- KEGG FOCAL ADHESION
- BIOCARTA RACCYCD PATHWAY

GF/MAPK
- BIOCARTA BCR PATHWAY
- BIOCARTA PTEN PATHWAY
- REACTOME SIGNALLING BY NGF
- BIOCARTA RACCYCD PATHWAY
- KEGG PHOSPHATIDYLINOSITOL SIGNALING SYSTEM
- KEGG INSULIN SIGNALING PATHWAY
- SIG PIP3 SIGNALING IN CARDIAC MYOCTES
- REACTOME CREB PHOPHORYLATION THROUGH THE ACTIVATION OF RAS
- BIOCARTA TNFR1 PATHWAY
- KEGG MAPK SIGNALING PATHWAY
- REACTOME NRAGE SIGNALS DEATH THROUGH JNK
- REACTOME TRKA SIGNALLING FROM THE PLASMA MEMBRANE
- ST PHOSPHOINOSITIDE 3 KINASE PATHWAY

Cancer
- KEGG SMALL CELL LUNG CANCER
- KEGG PANCREATIC CANCER
- KEGG PATHWAYS IN CANCER
- KEGG ENDOMETRIAL CANCER
- KEGG BASAL CELL CARCINOMA
- KEGG ACUTE MYELOID LEUKEMIA
- BIOCARTA P53HYPOXIA PATHWAY

Cell cycle
- KEGG SMALL CELL LUNG CANCER
- BIOCARTA RACCYCD PATHWAY
- BIOCARTA G2 PATHWAY
- SA G2 AND M PHASES
- BIOCARTA P53HYPOXIA PATHWAY

Other
- BIOCARTA BCR PATHWAY
- REACTOME SIGNALING IN IMMUNE SYSTEM
- KEGG FRUCTOSE AND MANNOSE METABOLISM
- BIOCARTA AGR PATHWAY
- KEGG NATURAL KILLER CELL MEDIATED CYTOTOXICITY
- REACTOME PLATELET AGGREGATION PLUG FORMATION
- REACTOME TRANSMISSION ACROSS CHEMICAL SYNAPSES
- KEGG METABOLIC PATHWAYS
- REACTOME HEMOSTASIS
- KEGG HOMOLOGOUS RECOMBINATION
- REACTOME LATE PHASE OF HIV LIFE CYCLE
- KEGG NEUROTROPHIN SIGNALING PATHWAY
- REACTOME METABOLISM OF AMINO ACIDS
- KEGG LONG TERM DEPRESSION
- KEGG HYPERTROPHIC CARDIOMYOPATHY HCM
- KEGG TYPE II DIABETES MELLITUS
- BIOCARTA ACH PATHWAY
- REACTOME INNATE IMMUNITY SIGNALING
- KEGG AMOEBIASIS
- BIOCARTA IL12 PATHWAY

Table S3: Relative band area of each protein in the gel quantified by ImageJ software.

Tissue Sample #		VTN	ITGA5	ITGA4	PTEN	P53	ITGA6	F-actin	ICAM2	FAK	β-actin
Prophylactic	1100263	1136	20044	2319	8317	10473	16818	98	13165	225	7300
	1101200	20160	15595	16324	14448	9780	14449	16278	8542	498	7300
	1106377	30929	5216	24701	48855	17795	21834	12724	16422	8802	7300
	1107863	15081	14433	51198	51243	28477	16436	31440	28282	8750	7300
	1111158	18149	12454	7194	15927	12325	21414	1889	9226	536	7300
	1202836	59	899	1974	9076	13396	11141	1157	12256	25436	7300
	1204812	1036	21428	1732	2038	30263	18663	54	4472	28769	7300
	1305982	13746	8663	147	5702	6155	11843	13422	7679	10231	7300
	1308228	17135	5590	9121	1568	4647	954	5067	9895	452	7300
	1310783	2112	18196	18262	17690	9296	12894	12655	13205	1067	7300
	1312767	108158	56922	7134	7918	46524	66464	309	51498	7864	7300
	1313347	29729	29424	7277	6790	12060	28699	0	20218	21619	7300
	1314445	20071	11395	19795	12946	11051	13212	13772	15798	17514	7300
	1315337	34925	27023	3465	7798	29994	11255	1237	22634	22802	7300
	1104671	987	2638	5610	8182	8326	4860	13152	10812	1343	7300
	1214311	15533	8292	4426	6438	6715	4579	2206	1471	0	7300
	1304859	6114	4959	225	6699	5036	8477	4864	2593	4049	7300
	1306442	10811	7960	8567	8848	4322	12107	5429	4743	0	7300
	1306784	918	5953	7596	5744	6777	13929	5523	2925	3040	7300
	1311366	5790	5982	1517	1857	15213	8306	270	5261	5869	7300
	2011-1259	521	7770	3150	4323	20492	3217	2319	3466	5666	7300
	2011-1286	3073	6696	10866	11902	7498	9260	6422	8750	1302	7300
Normal	1306291	55	25550	19817	19235	10203	13195	5120	19533	9583	7300
	1307090	2662	5153	170	566	12392	23240	123	4971	21077	7300
	1311144	17651	8679	5203	9993	15505	13330	2047	11265	10103	7300
	1305256	4387	17015	87	12811	8816	12885	9477	8662	12453	7300
	1305256	603	48122	40752	25688	35197	32262	14705	25175	16946	7300
	1305429	2057	11483	1921	17146	953	14314	26663	12574	10310	7300
	1310673	389	1110	18766	14509	15674	13320	3764	9638	1130	7300
	1308946	5025	20119	26421	30573	28567	29277	16661	25971	23125	7300
	1314284	844	11925	29685	27620	9776	18087	28125	18909	13851	7300
	1108681	557	7717	727	13238	6483	2896	11569	5035	5604	7300
	1301318	742	8341	1532	11023	13685	3989	12453	7068	9940	7300
	1109190	329	6884	143	11325	6849	944	9087	3256	5662	7300
	1306291	17634	8159	18283	16419	16058	14835	10763	17070	21549	7300
	1217389	793	11401	1946	13492	5017	1562	11605	7145	0	7300
	1308946	5681	37813	48037	42979	38247	70346	34387	32181	14187	7300
	1305256	1508	10101	7638	8695	6825	9119	7961	9034	5011	7300
Average of FBC		16190	13524	9664	12014	14392	15037	6831	12423	7992	7300
Average of Control		3807	14973	13820	17207	14390	17100	12782	13593	11283	7300
p value		**0.04**	0.73	0.35	0.19	1.00	0.67	**0.04**	0.73	0.24	0.96

Figures

Figure S1: Distribution of ages for patients in the Utah cohort.

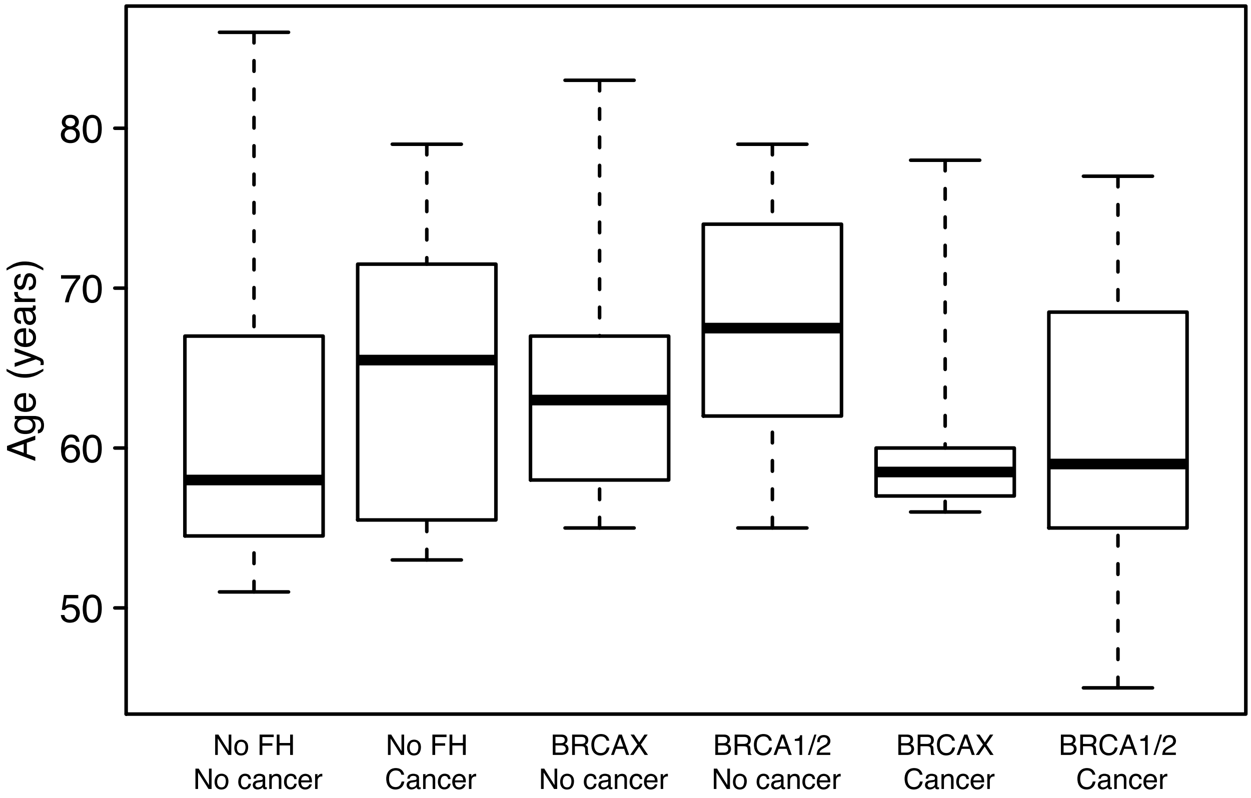

Figure S2: Summary of variant effects on protein function. Most potentially pathogenic variants were non-synonymous substitutions. However, many other types of variant effect were also observed.

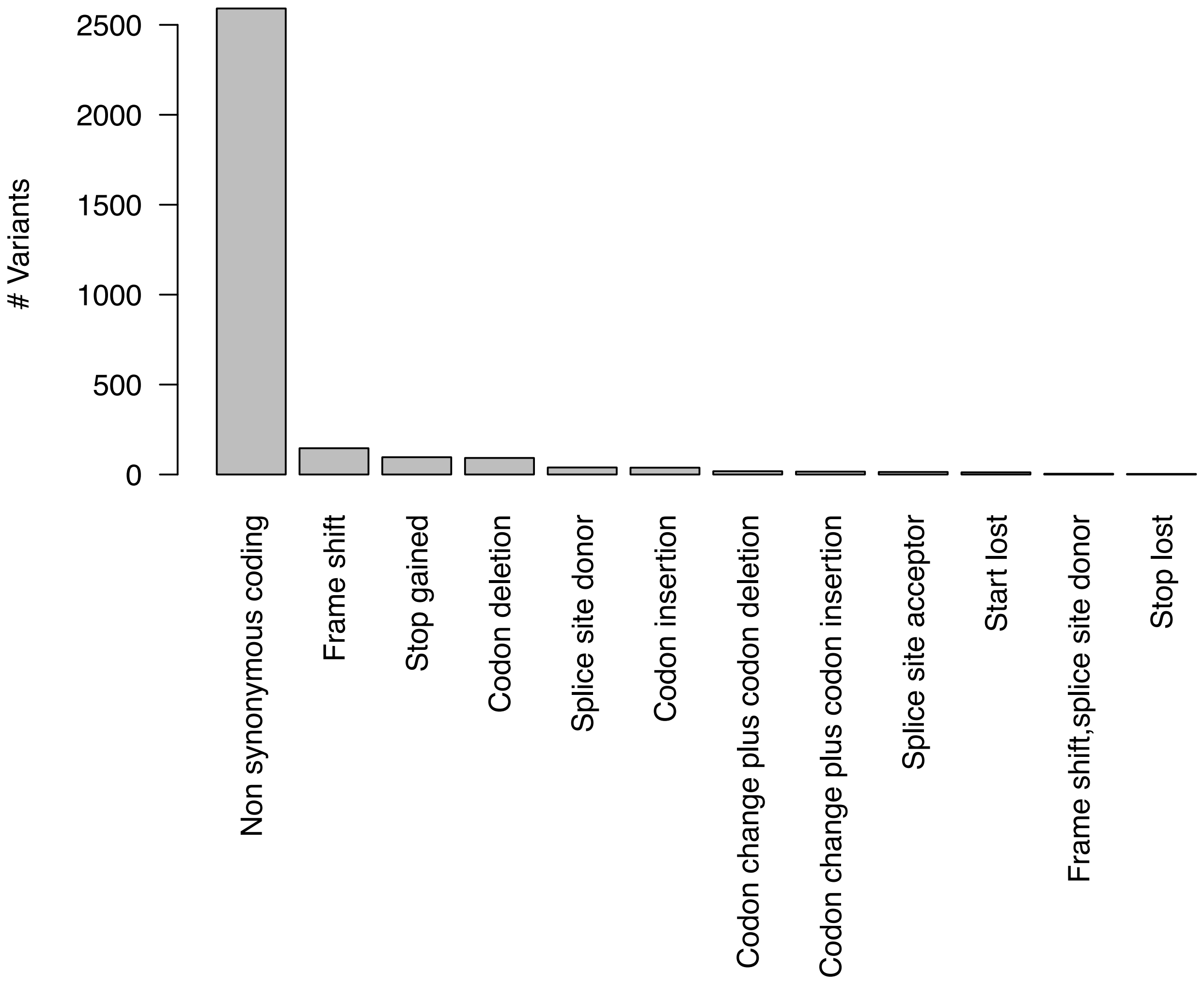

Figure S3: Summary of DNA alterations that resulted from potentially pathogenic variants. Single-nucleotide substitutions exhibited a non-uniform distribution; G-to-A and C-to-T transitions were most common. Deletions were more common than insertions.

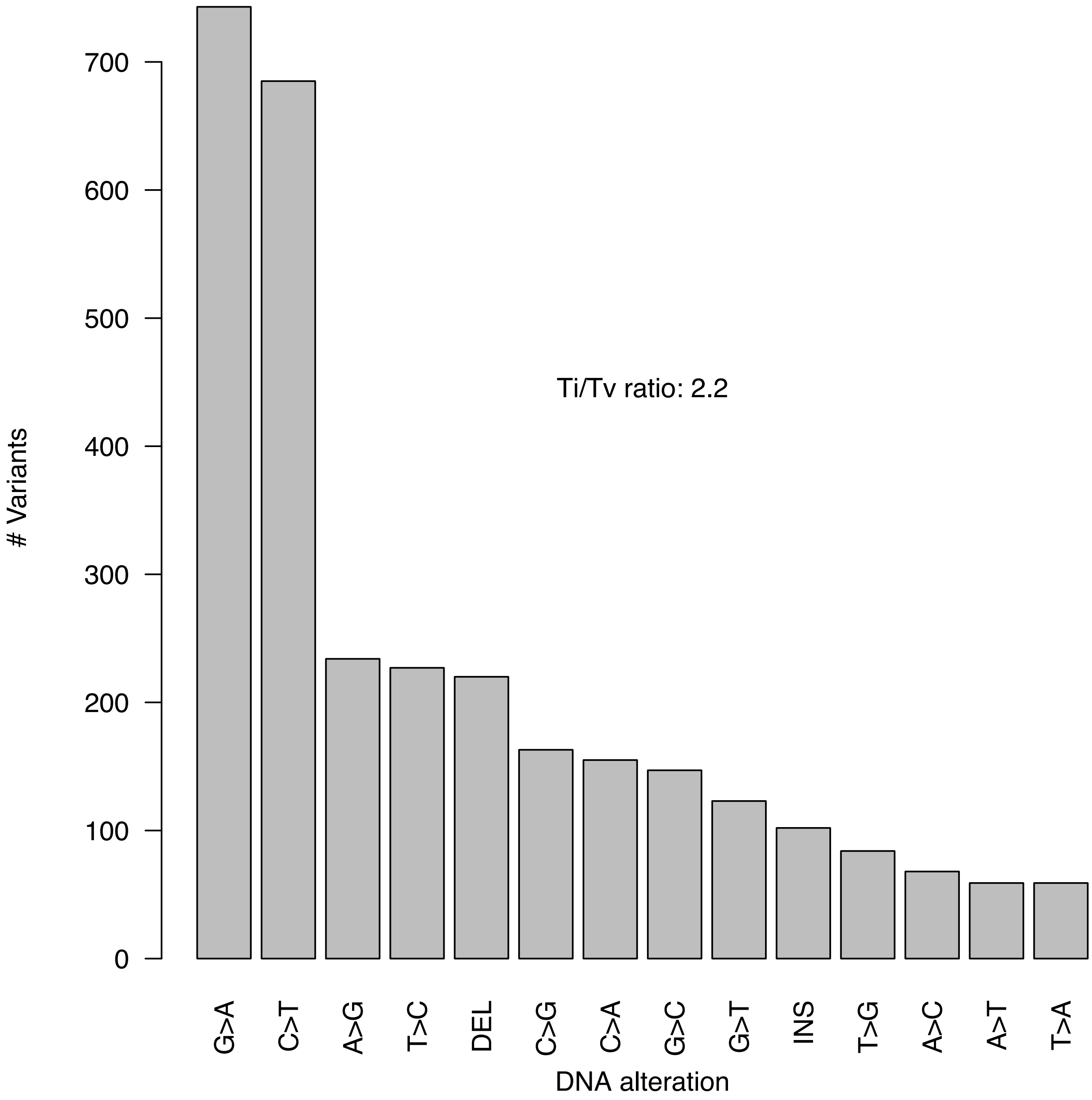

Figure S4: Heatmaps showing median expression levels for Utah and Ontario women who developed FBC and for women who did not, separated according to BRCA1 and BRCA2 mutation status, for two key pathways. Only genes that exhibited a consistent fold change across the cohorts are shown.

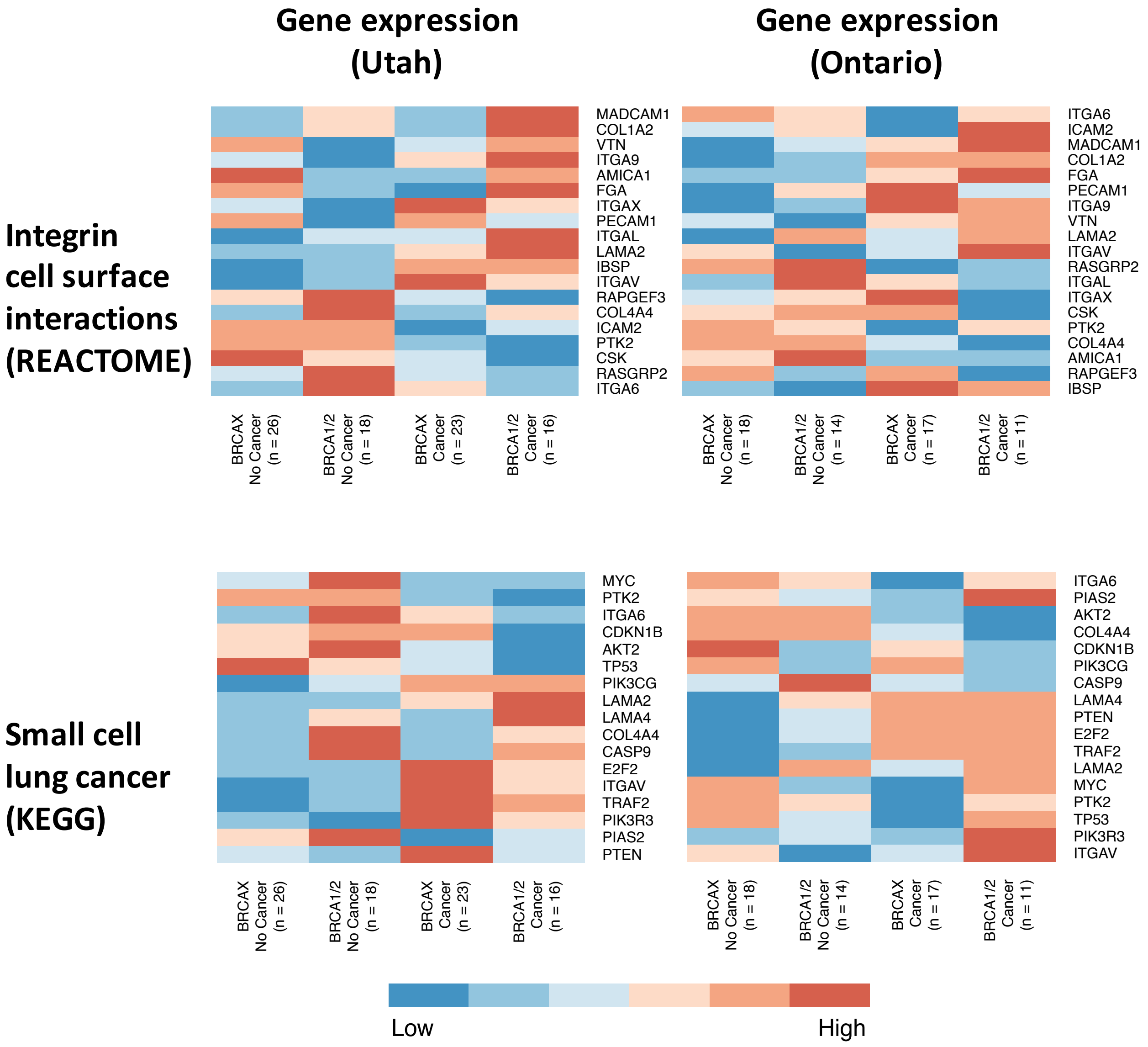

Figure S5: Per-sample DNA variants observed in two key pathways. Black dots indicate samples that carried a likely pathogenic in the genes that are shown. Only genes for which at least one variant was observed are shown.

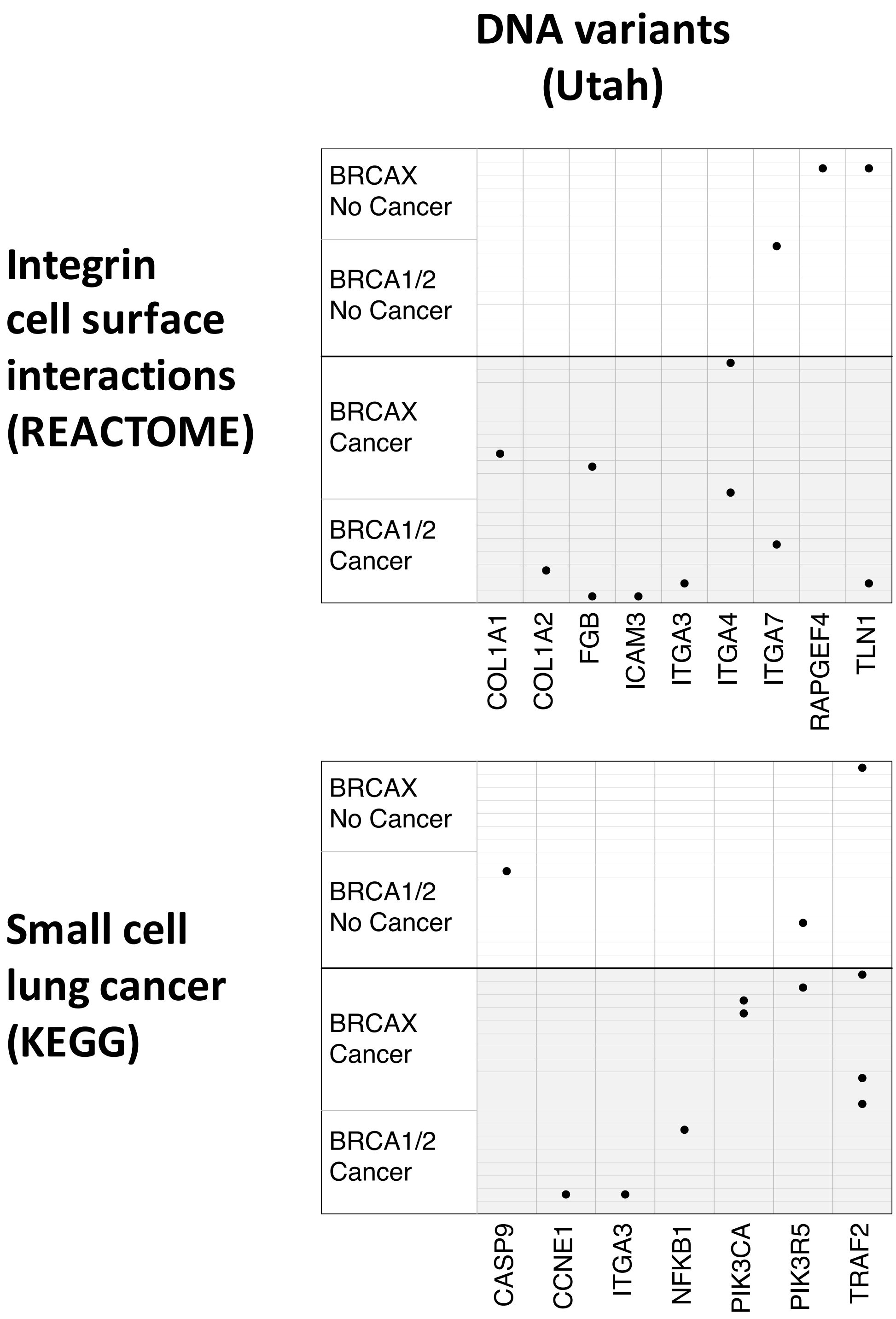

Supplement: Supplementary file 1 — Appendix [file MSB-12-860-s001.pdf]
