# Supplementary material for: Integrative analyses reveal signaling pathways underlying familial breast cancer susceptibility
Source: Mol Syst Biol. 2016 Mar 11;12(3):860. doi: 10.15252/msb.20156506 (PMC4812528; doi:10.15252/msb.20156506)
Supplement: Supplementary file 2 — Expanded View Figures PDF [file MSB-12-860-s002.pdf]

Expanded View Figures

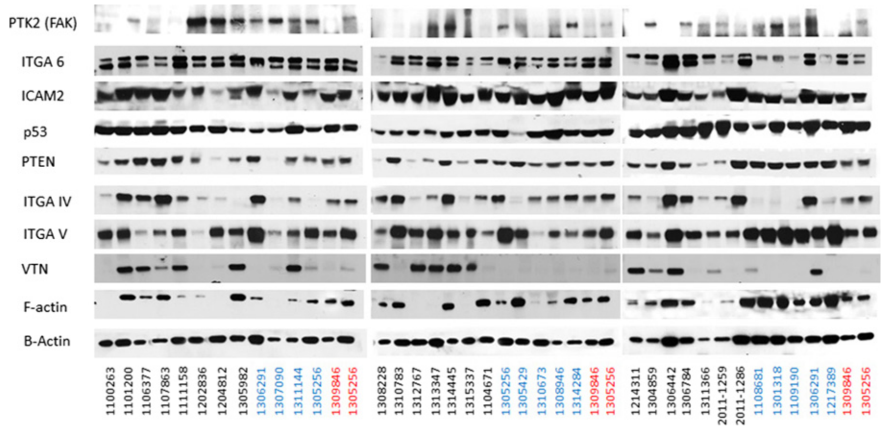

Figure EV1. Western blots of selected proteins.
Cell lysates obtained from cultures of primary mammary epithelial cells for women having normal breast reduction (control) or prophylactic breast reduction (high-risk women) surgeries were immunoblotted with FAK (PTK2), ITGA6, ICAM2, p53, PTEN, ITGA IV, ITGA V, VTN, F-actin, and β-actin (loading control). Two lysates were loaded to each gel to compare between blots. Sample names shown in black are from high-risk women; sample names shown in blue are from normal breast reduction samples; sample names shown in red are from breast reduction normal controls placed on every gene to serve as internal loading/processing controls.

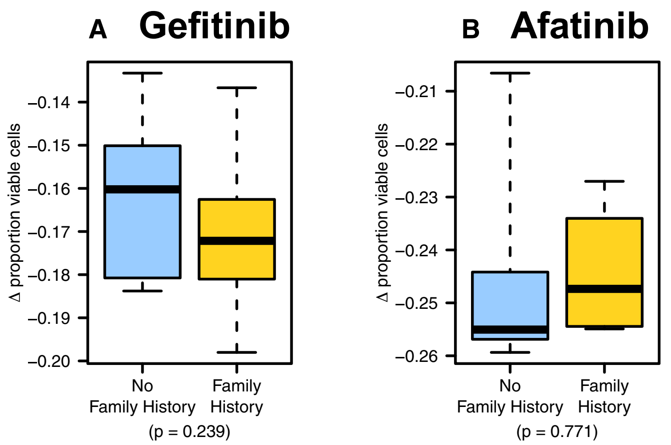

Figure EV2. Gefitinib and afatinib assay results.
A, B Primary breast cells were treated with the (A) EGFR inhibitor gefitinib and (B) tyrosine kinase inhibitor afatinib. Similar responses were observed for women who had a family history of breast cancer and for women who did not have a family history of breast cancer. The boxes represent the the interquartile range of the respective values. The whiskers extend the the most extreme data points.

© 2016 The Authors

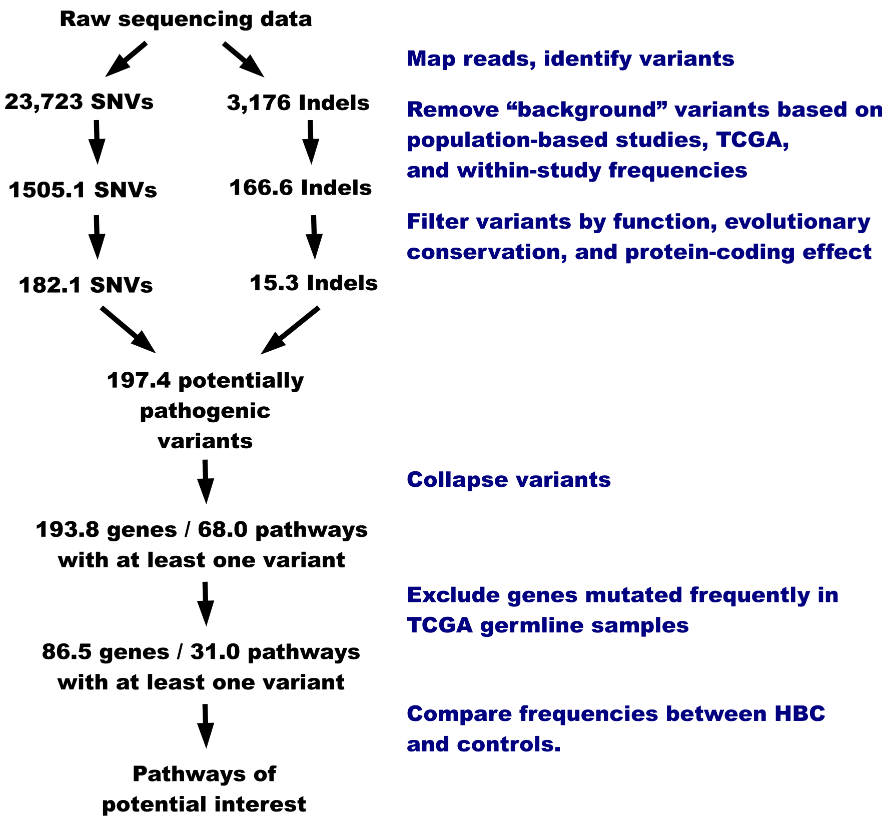

Figure EV3. Overview of criteria used to filter exome-sequencing variants.
Variants were filtered based on frequency, location within protein-coding regions, conservation, and effect on protein sequence. Variants were also collapsed to gene-level values before pathway-level comparisons were performed. All statistics listed on this diagram are per sample.

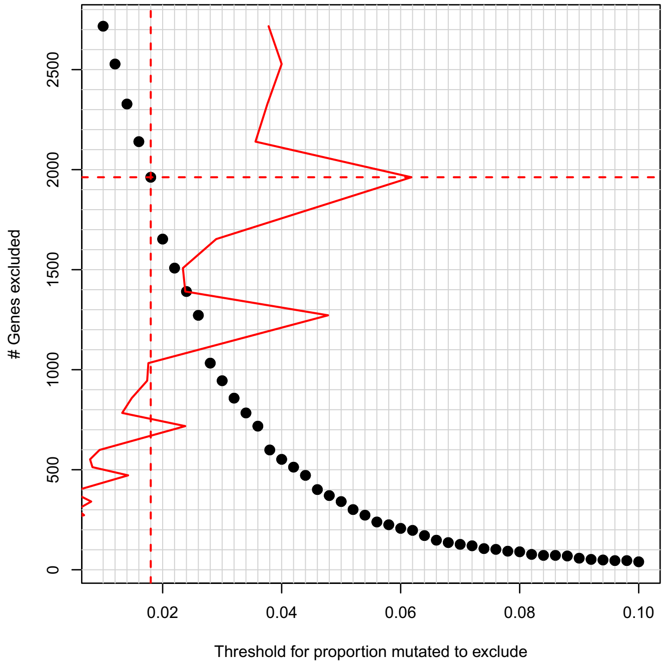

Figure EV4. Selection of threshold used to filter frequently mutated genes.
Genes that were mutated in a relatively high number of germline samples in TCGA were excluded from the pathway-level mutation analyses. We calculated the number of genes that would be excluded for thresholds ranging between 0.2% and 10%. A threshold of 1.8% was selected based on the maximal difference in number of excluded genes.

© 2016 The Authors

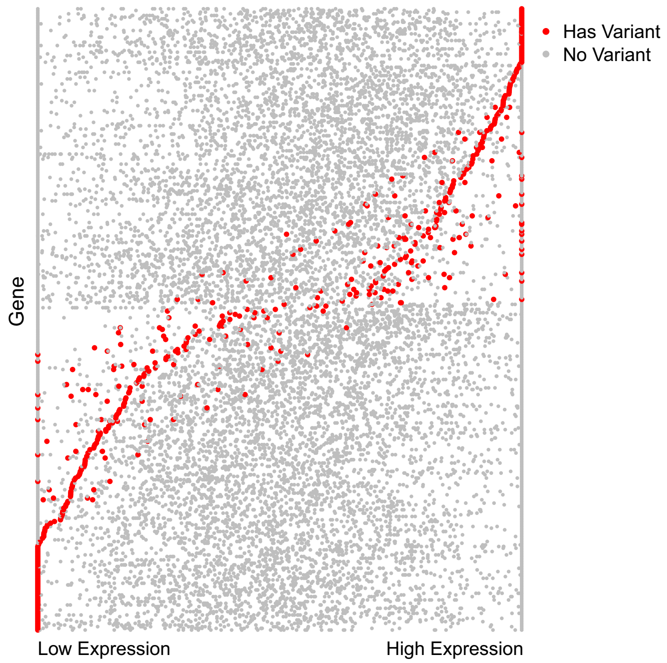

Figure EV5. Relationship between variant status and gene expression for 34 samples profiled using gene expression microarrays and exome sequencing.
We sought to identify genes whose expression levels correlated strongly with mutation status. This figure shows data for 373 genes that exhibited the strongest association between the presence of one or more potentially pathogenic variants and expression of the same gene. Red dots indicate samples that carried a mutation within a given gene. Gray dots indicate samples that did not carry a mutation within the same gene. In many cases, germline variants are correlated with a considerable increase or decrease of expression levels for the same gene. Expression values for each gene are standardized to a consistent scale for illustration purposes.

© 2016 The Authors
