# Supplementary figures and images for: Integrative analyses reveal signaling pathways underlying familial breast cancer susceptibility
Source: Mol Syst Biol. 2016 Mar 11;12(3):860. doi: 10.15252/msb.20156506 (PMC4812528; doi:10.15252/msb.20156506)

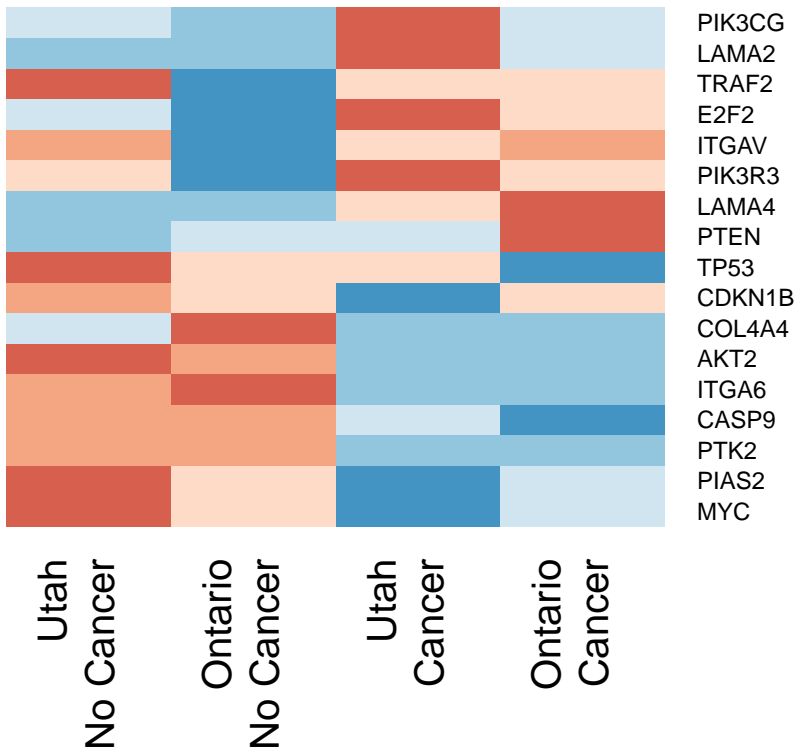

Supplement: Supplementary file 20 — Computer Code EV2 [file MSB-12-860-s020.zip › MSB-15-6506R_Computer_Code_EV2/BCRiskPathways_Notebook/Figures/Figure_2_Expr_SCLC_Utah.pdf]

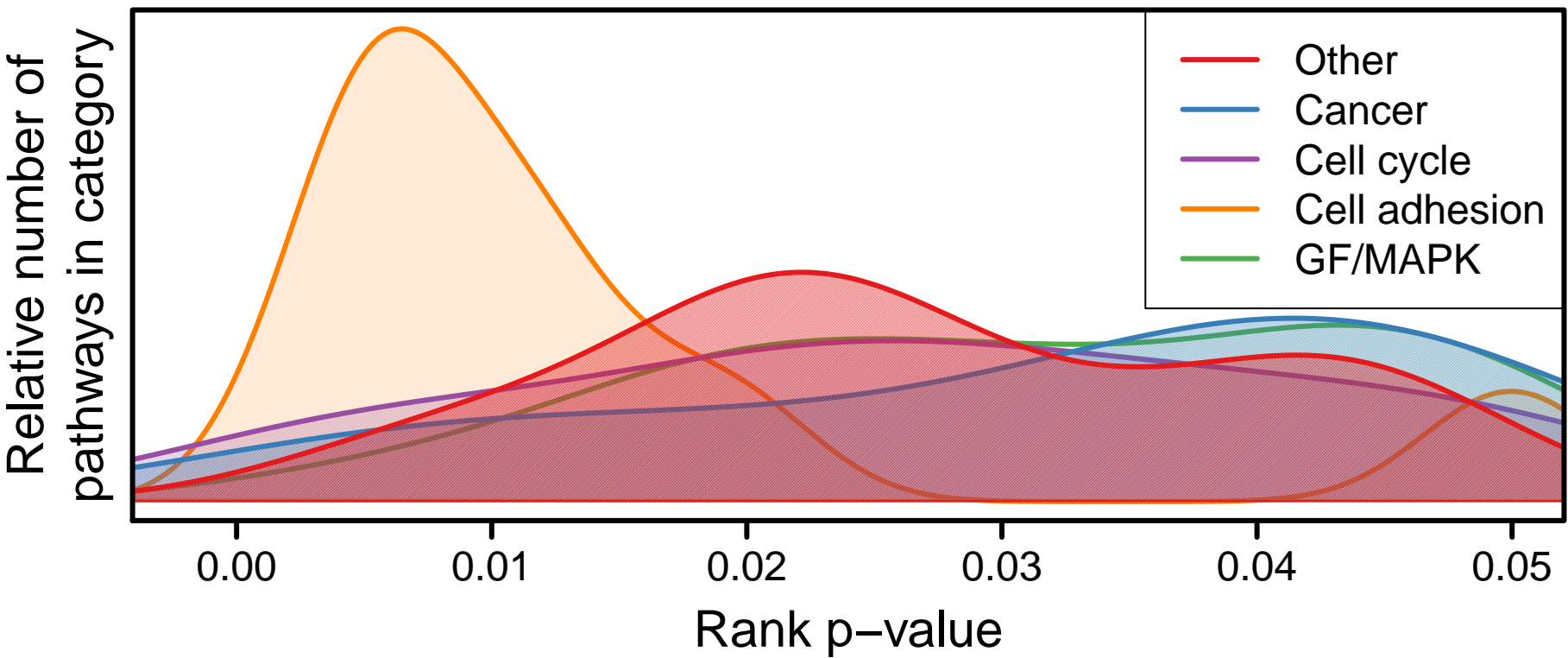

Supplement: Supplementary file 20 — Computer Code EV2 [file MSB-12-860-s020.zip › MSB-15-6506R_Computer_Code_EV2/BCRiskPathways_Notebook/Figures/Figure_2A.pdf]

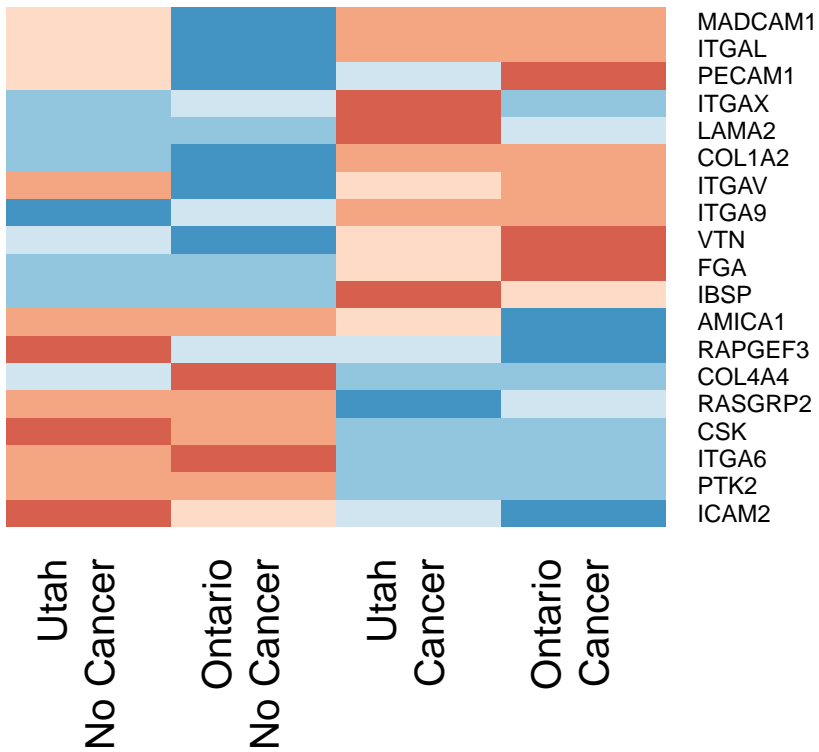

Supplement: Supplementary file 20 — Computer Code EV2 [file MSB-12-860-s020.zip › MSB-15-6506R_Computer_Code_EV2/BCRiskPathways_Notebook/Figures/Figure_2B_Integrin.pdf]

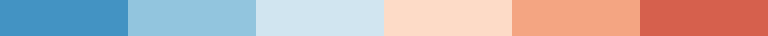

Low

High

Supplement: Supplementary file 20 — Computer Code EV2 [file MSB-12-860-s020.zip › MSB-15-6506R_Computer_Code_EV2/BCRiskPathways_Notebook/Figures/Figure_2B_Legend.pdf]

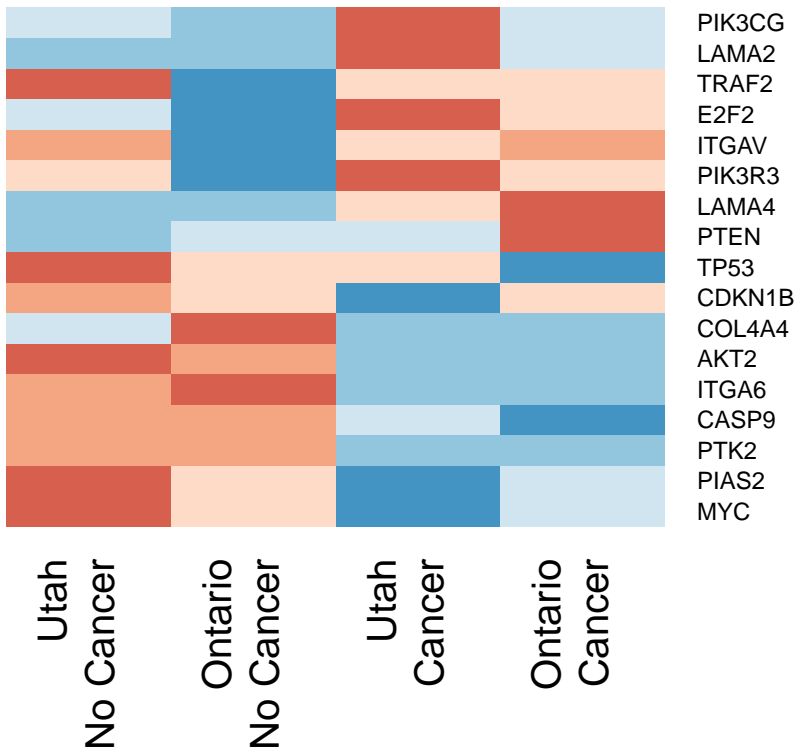

Supplement: Supplementary file 20 — Computer Code EV2 [file MSB-12-860-s020.zip › MSB-15-6506R_Computer_Code_EV2/BCRiskPathways_Notebook/Figures/Figure_2B_SCLC.pdf]

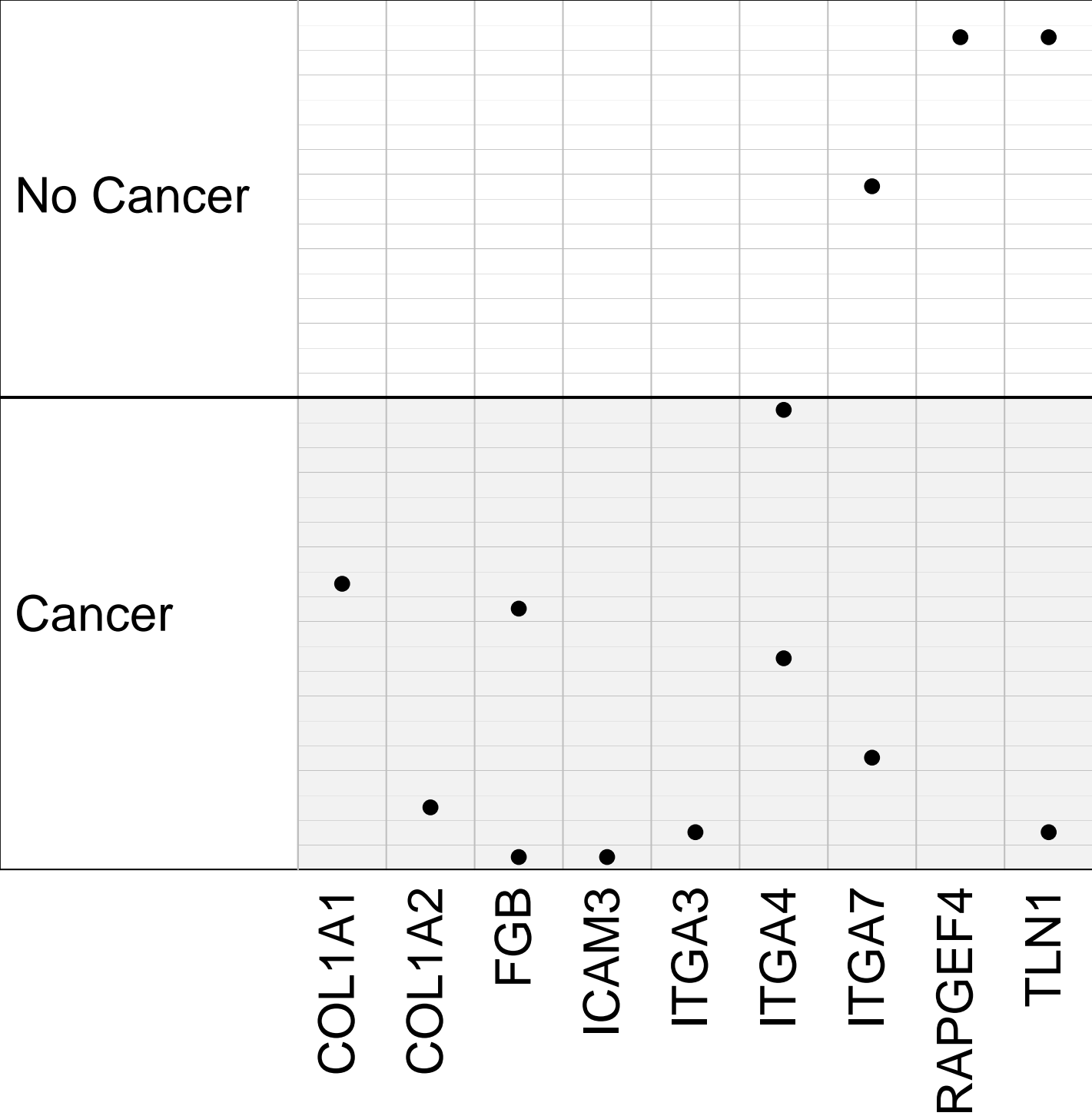

Supplement: Supplementary file 20 — Computer Code EV2 [file MSB-12-860-s020.zip › MSB-15-6506R_Computer_Code_EV2/BCRiskPathways_Notebook/Figures/Figure_2C_Integrin.pdf]

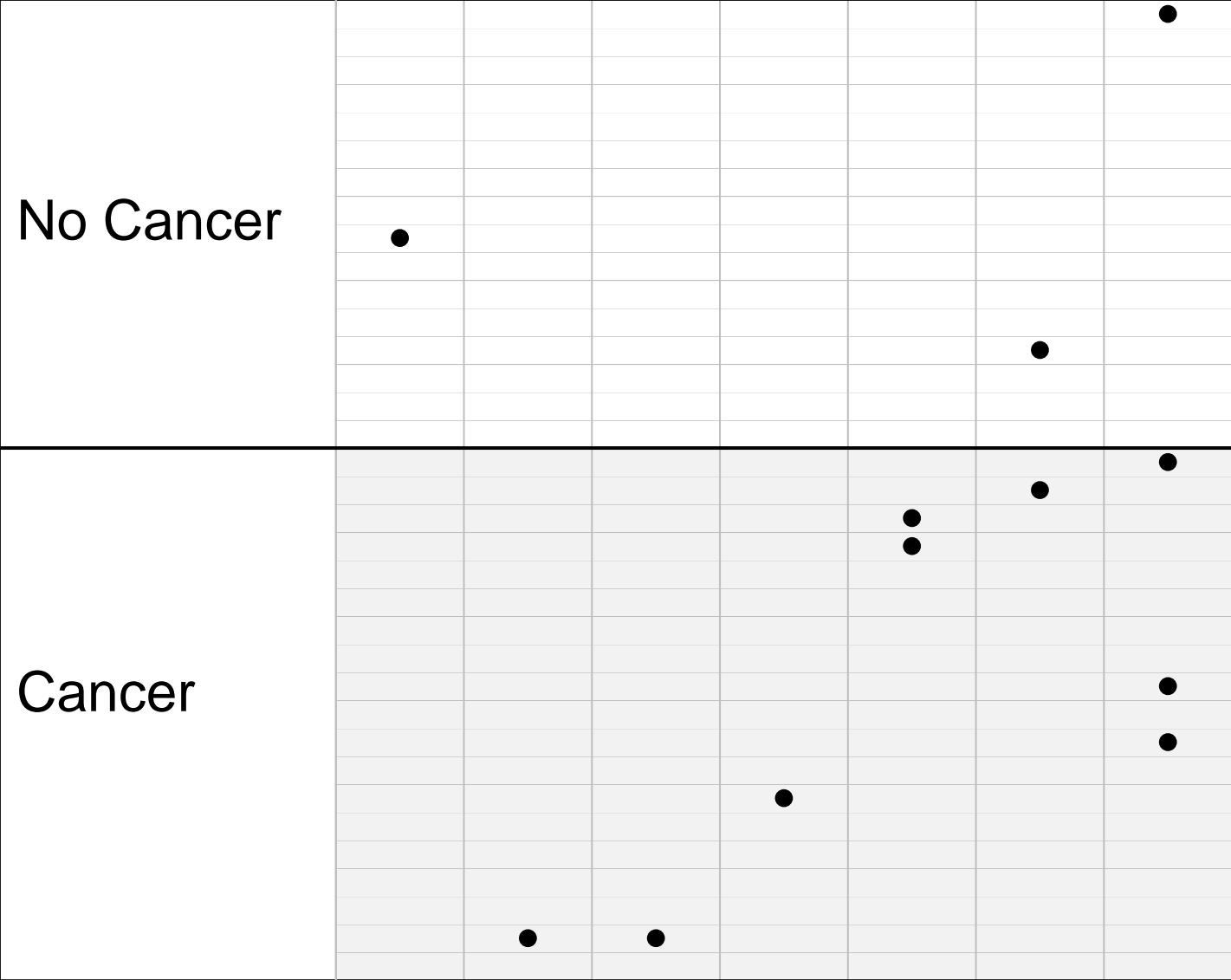

No Cancer

Cancer

CASP9

CCNE1

ITGA3

NFKB1

PIK3CA

PIK3R5

TRAF2

Supplement: Supplementary file 20 — Computer Code EV2 [file MSB-12-860-s020.zip › MSB-15-6506R_Computer_Code_EV2/BCRiskPathways_Notebook/Figures/Figure_2C_SCLC.pdf]

Genomic model score

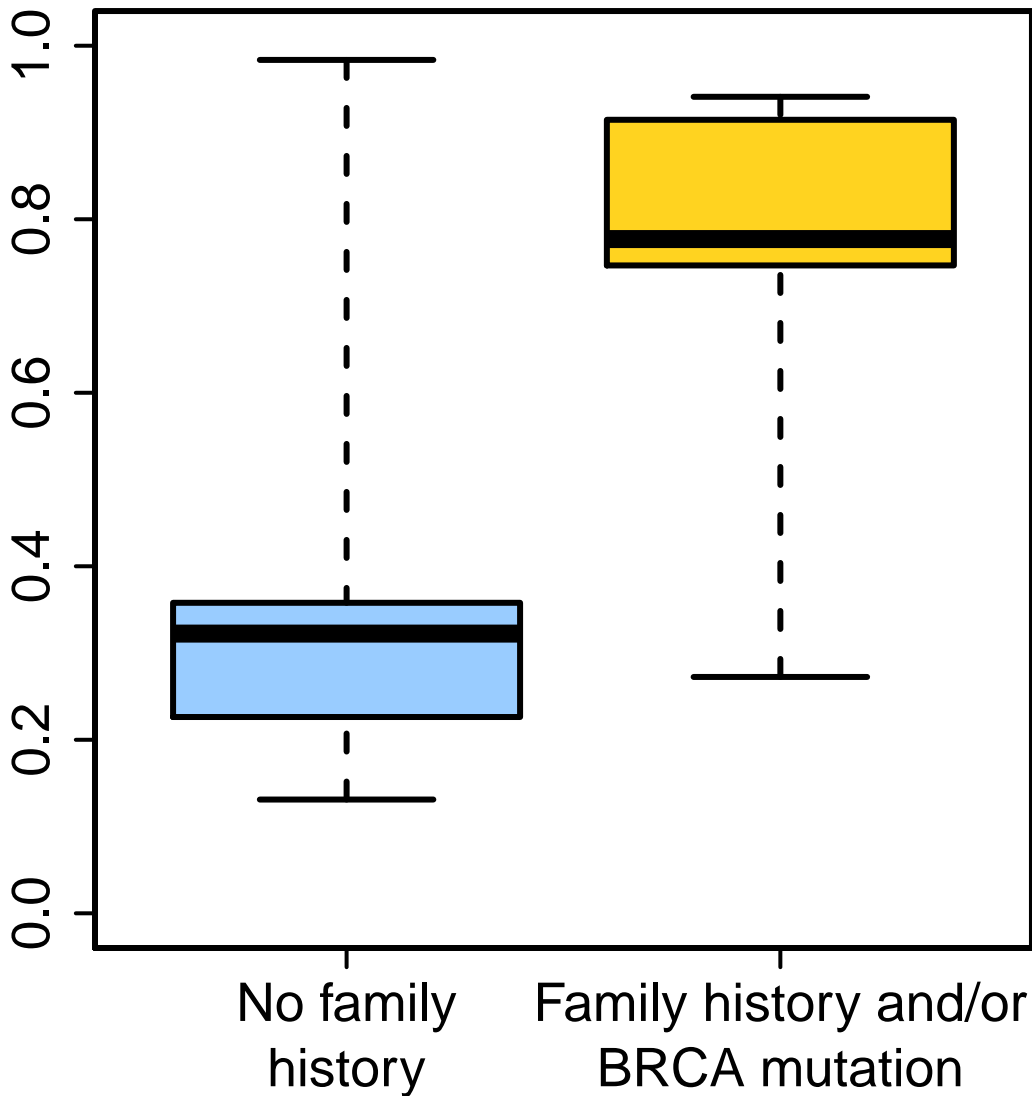

Supplement: Supplementary file 20 — Computer Code EV2 [file MSB-12-860-s020.zip › MSB-15-6506R_Computer_Code_EV2/BCRiskPathways_Notebook/Figures/Figure_3A1.pdf]

Genomic model score

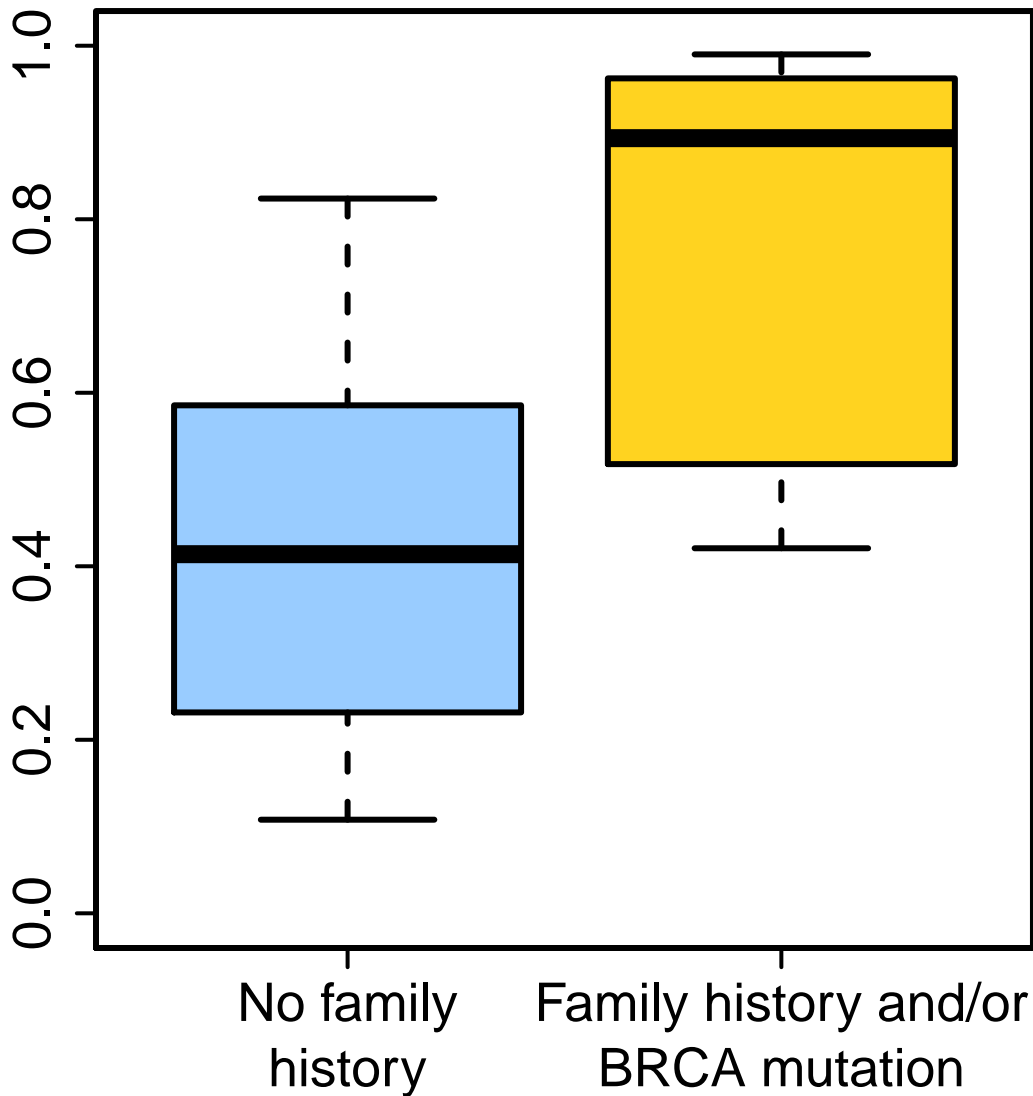

Supplement: Supplementary file 20 — Computer Code EV2 [file MSB-12-860-s020.zip › MSB-15-6506R_Computer_Code_EV2/BCRiskPathways_Notebook/Figures/Figure_3A2.pdf]

Genomic model score

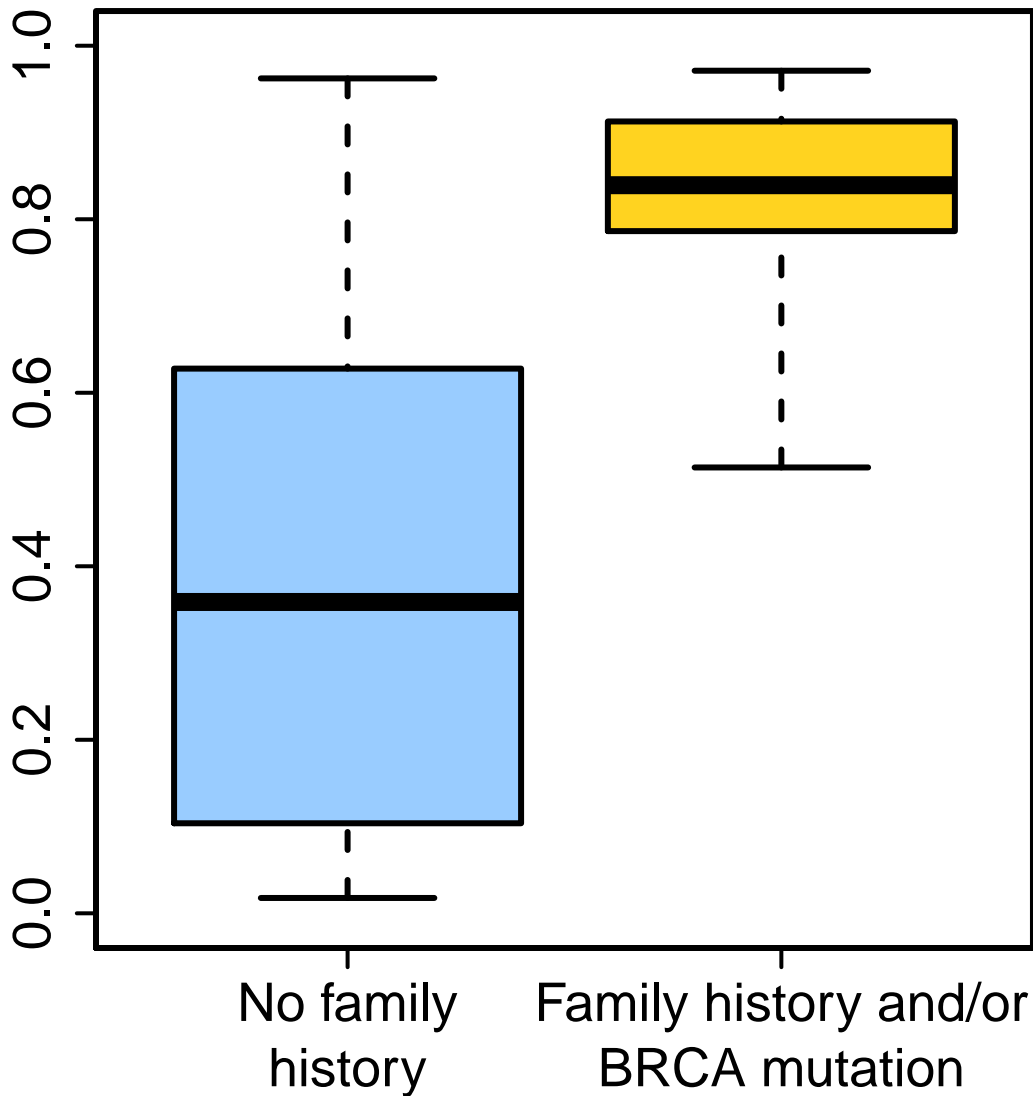

Supplement: Supplementary file 20 — Computer Code EV2 [file MSB-12-860-s020.zip › MSB-15-6506R_Computer_Code_EV2/BCRiskPathways_Notebook/Figures/Figure_3B1.pdf]

Genomic model score

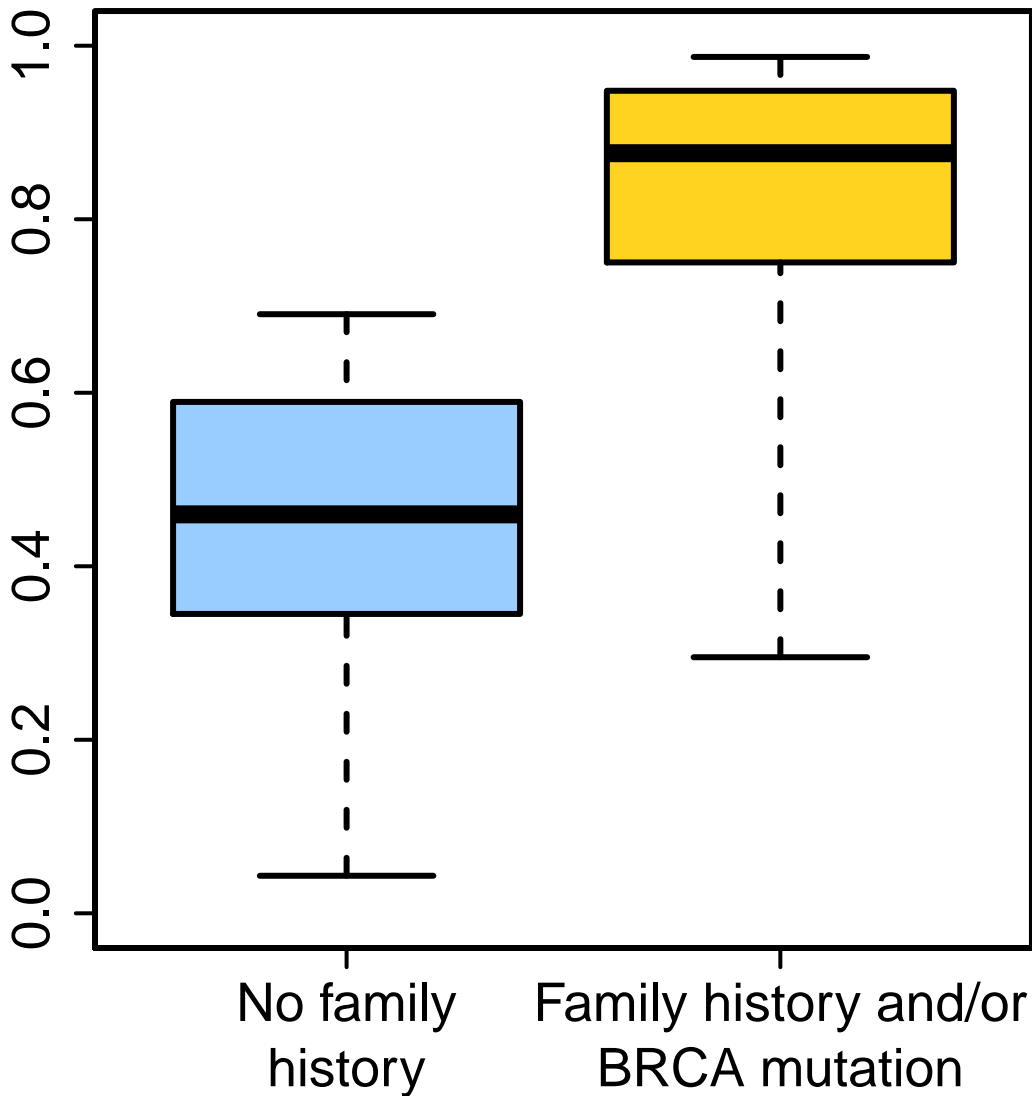

Supplement: Supplementary file 20 — Computer Code EV2 [file MSB-12-860-s020.zip › MSB-15-6506R_Computer_Code_EV2/BCRiskPathways_Notebook/Figures/Figure_3B2.pdf]

# % cell area per nucleus

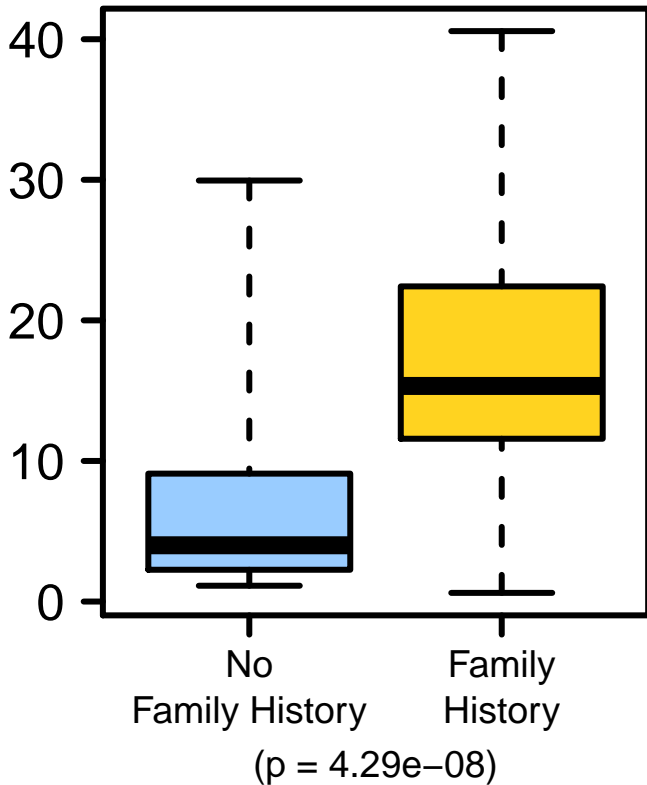

Supplement: Supplementary file 20 — Computer Code EV2 [file MSB-12-860-s020.zip › MSB-15-6506R_Computer_Code_EV2/BCRiskPathways_Notebook/Figures/Figure_4B1.pdf]

# Phalloidin per cell area

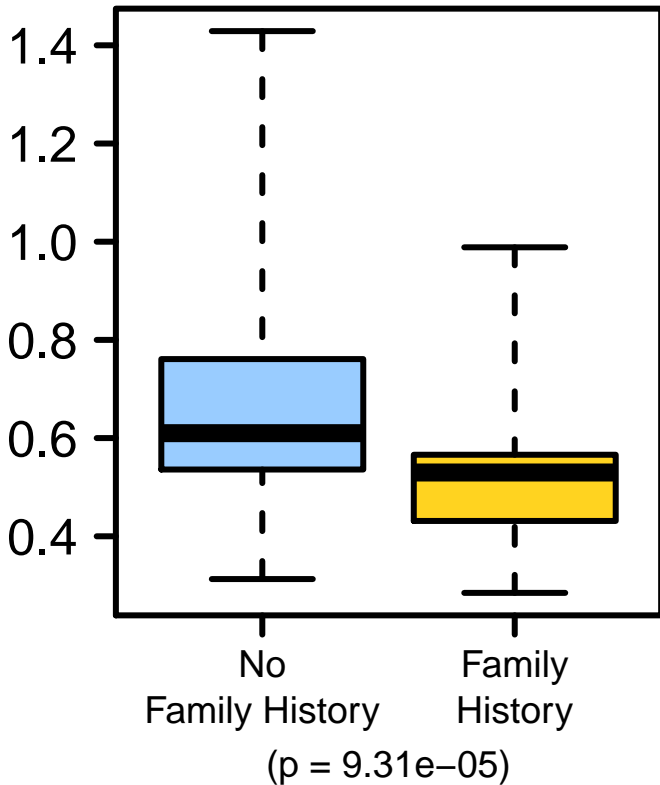

Supplement: Supplementary file 20 — Computer Code EV2 [file MSB-12-860-s020.zip › MSB-15-6506R_Computer_Code_EV2/BCRiskPathways_Notebook/Figures/Figure_4B2.pdf]

## Vinculin per cell area

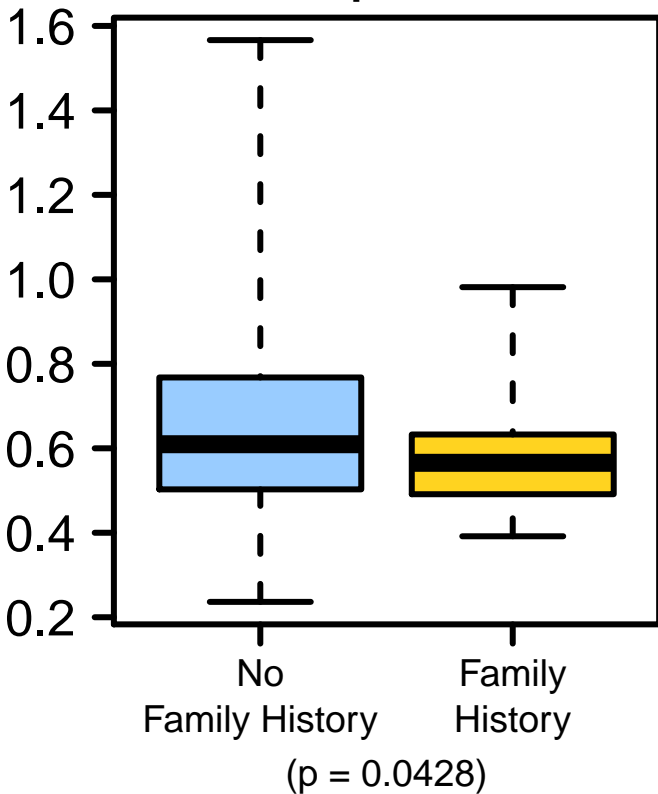

Supplement: Supplementary file 20 — Computer Code EV2 [file MSB-12-860-s020.zip › MSB-15-6506R_Computer_Code_EV2/BCRiskPathways_Notebook/Figures/Figure_4B3.pdf]

Relative adhesion

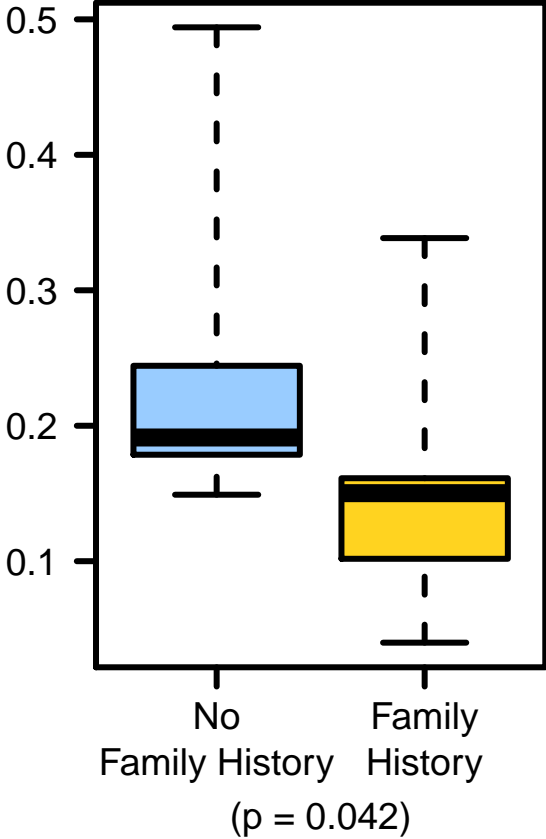

Supplement: Supplementary file 20 — Computer Code EV2 [file MSB-12-860-s020.zip › MSB-15-6506R_Computer_Code_EV2/BCRiskPathways_Notebook/Figures/Figure_5A.pdf]

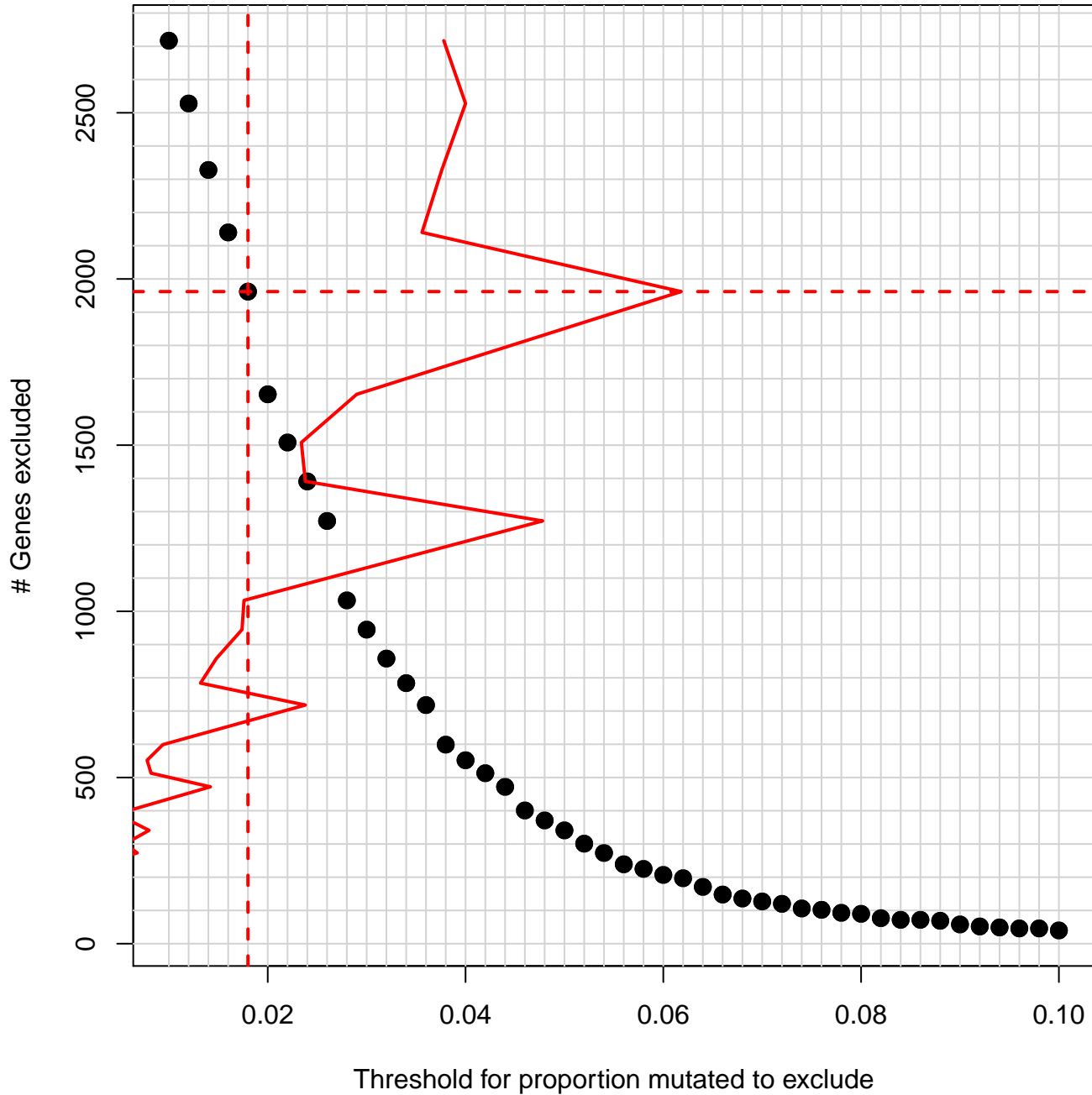

Supplement: Supplementary file 20 — Computer Code EV2 [file MSB-12-860-s020.zip › MSB-15-6506R_Computer_Code_EV2/BCRiskPathways_Notebook/Figures/Figure_EV2.pdf]

Gene

- Has Variant
- No Variant

Low Expression

High Expression

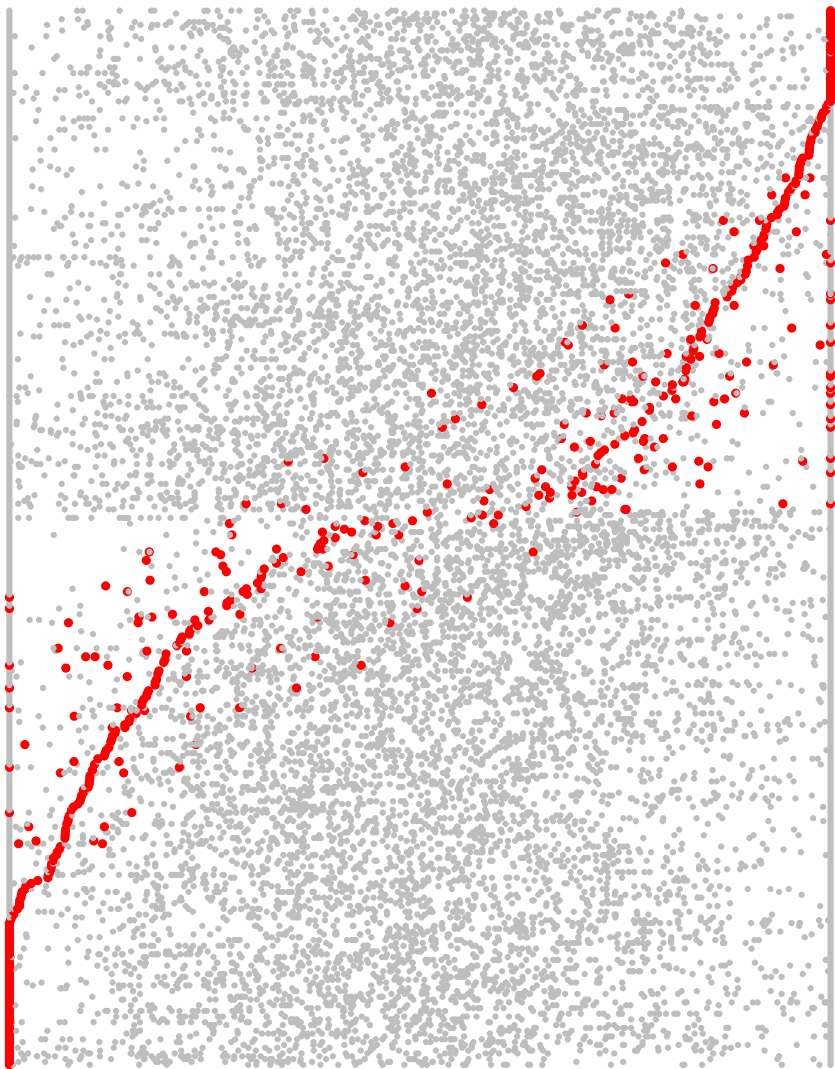

Supplement: Supplementary file 20 — Computer Code EV2 [file MSB-12-860-s020.zip › MSB-15-6506R_Computer_Code_EV2/BCRiskPathways_Notebook/Figures/Figure_EV3.pdf]

$\Delta$  proportion viable cells

-0.14  
-0.15  
-0.16  
-0.17  
-0.18  
-0.19  
-0.20

No  
Family History

Family  
History

( $p = 0.239$ )

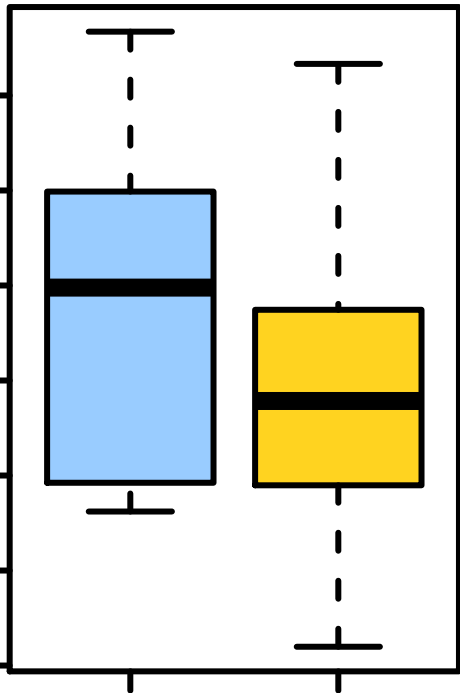

Supplement: Supplementary file 20 — Computer Code EV2 [file MSB-12-860-s020.zip › MSB-15-6506R_Computer_Code_EV2/BCRiskPathways_Notebook/Figures/Figure_EV5A.pdf]

$\Delta$  proportion viable cells

-0.21

-0.22

-0.23

-0.24

-0.25

-0.26

No

Family History

Family

History

( $p = 0.771$ )

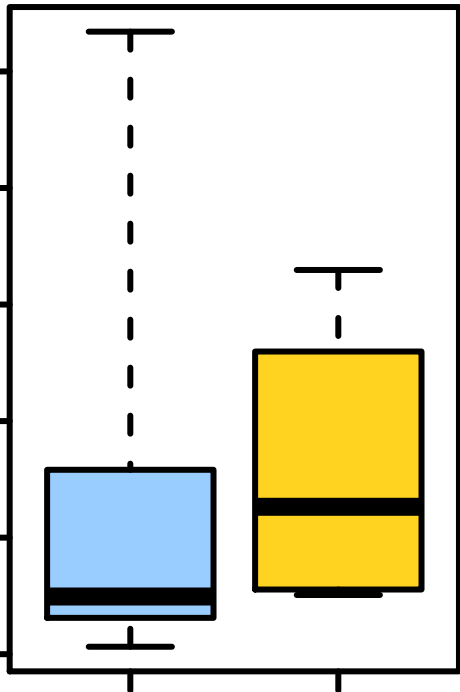

Supplement: Supplementary file 20 — Computer Code EV2 [file MSB-12-860-s020.zip › MSB-15-6506R_Computer_Code_EV2/BCRiskPathways_Notebook/Figures/Figure_EV5B.pdf]

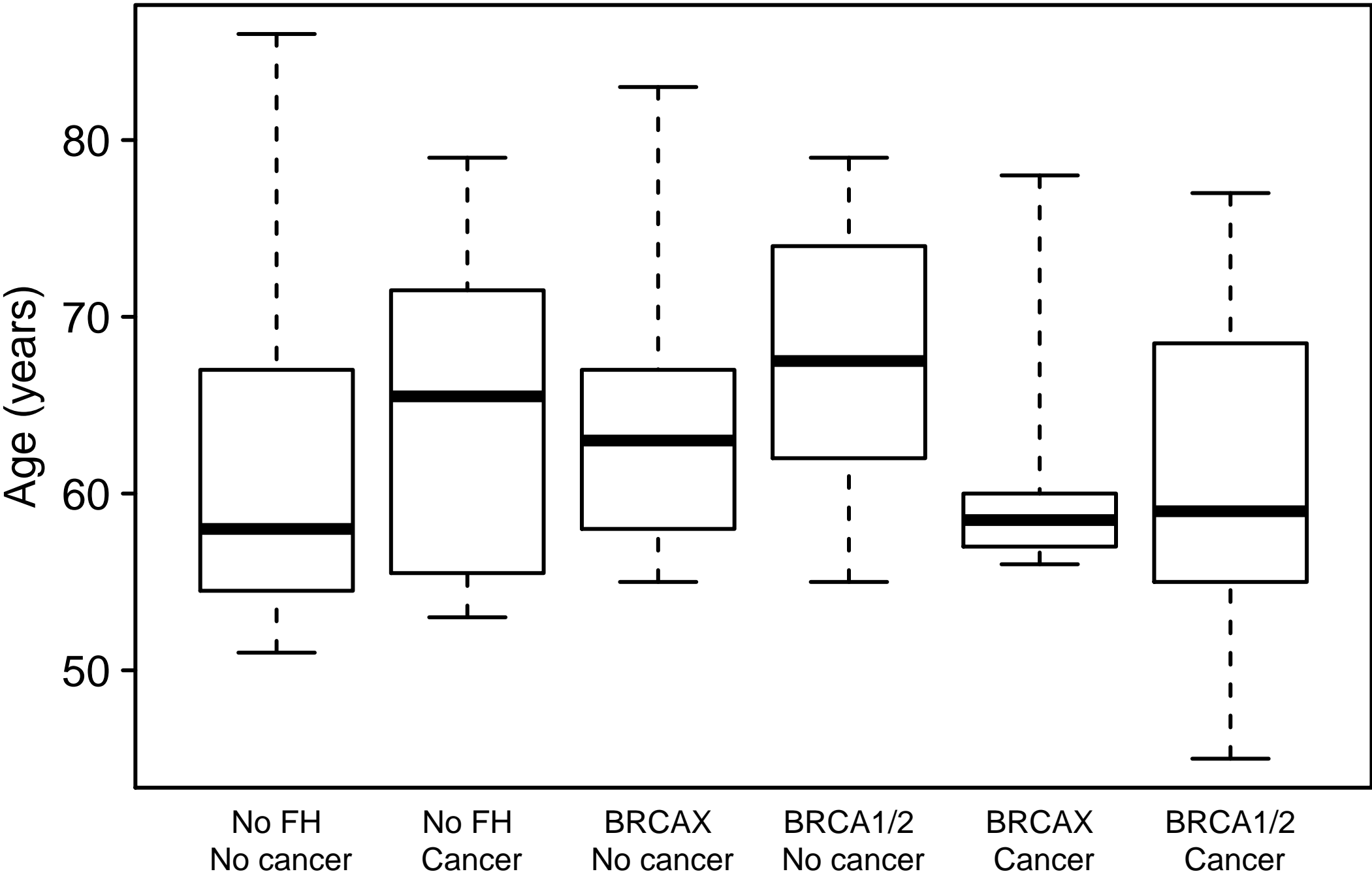

Supplement: Supplementary file 20 — Computer Code EV2 [file MSB-12-860-s020.zip › MSB-15-6506R_Computer_Code_EV2/BCRiskPathways_Notebook/Figures/Figure_S1.pdf]

# Variants

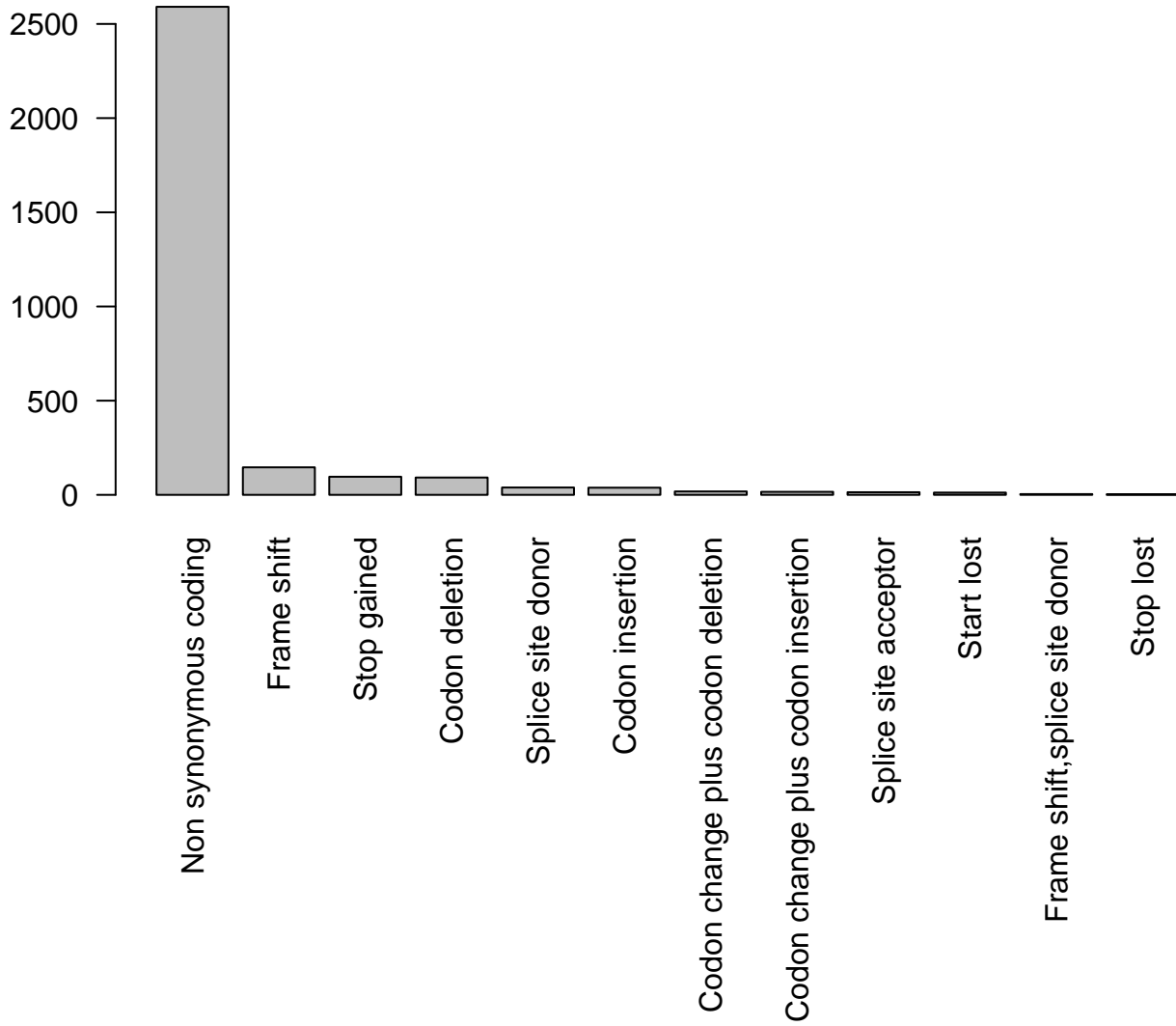

Supplement: Supplementary file 20 — Computer Code EV2 [file MSB-12-860-s020.zip › MSB-15-6506R_Computer_Code_EV2/BCRiskPathways_Notebook/Figures/Figure_S2.pdf]

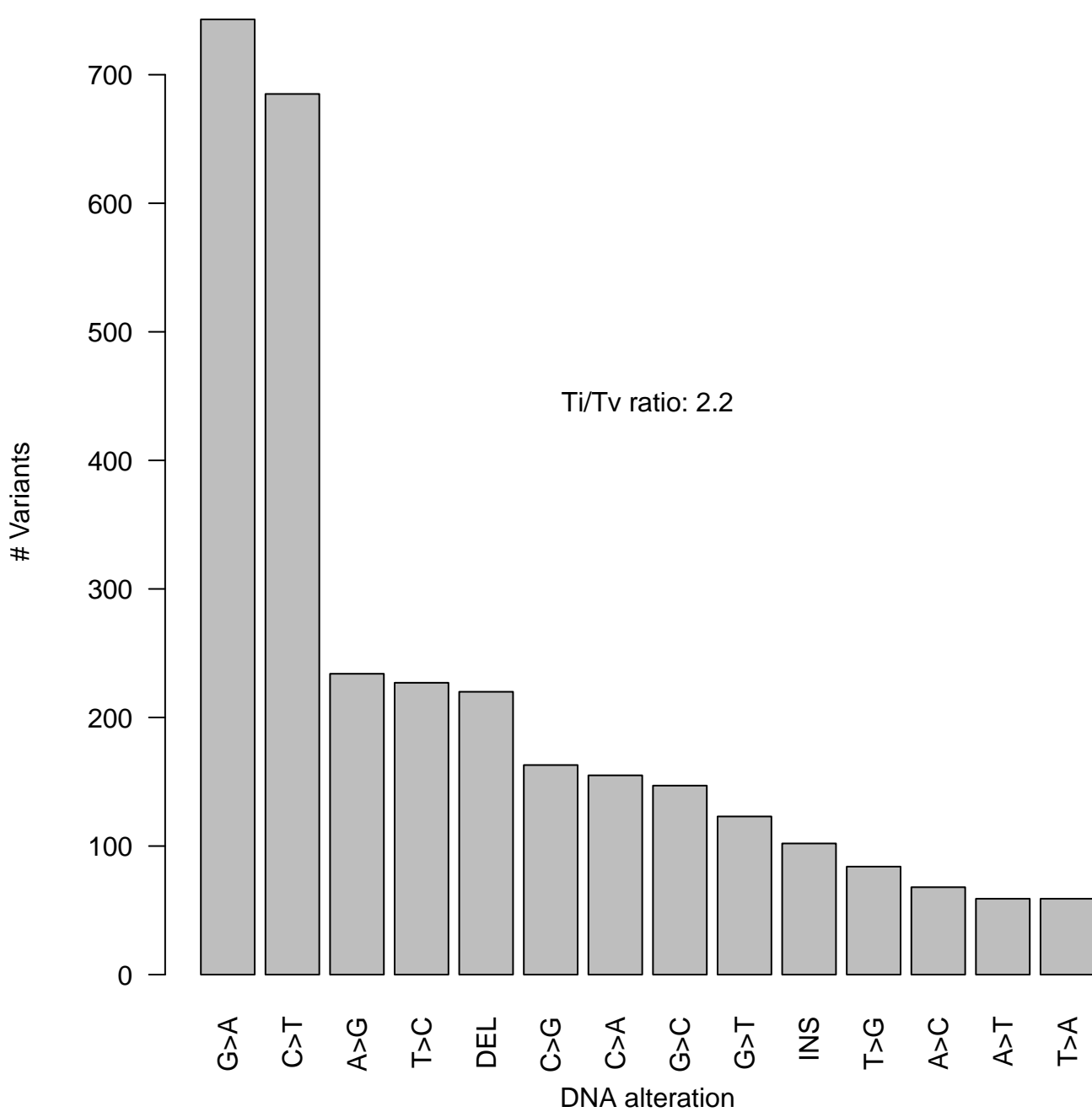

Supplement: Supplementary file 20 — Computer Code EV2 [file MSB-12-860-s020.zip › MSB-15-6506R_Computer_Code_EV2/BCRiskPathways_Notebook/Figures/Figure_S3.pdf]

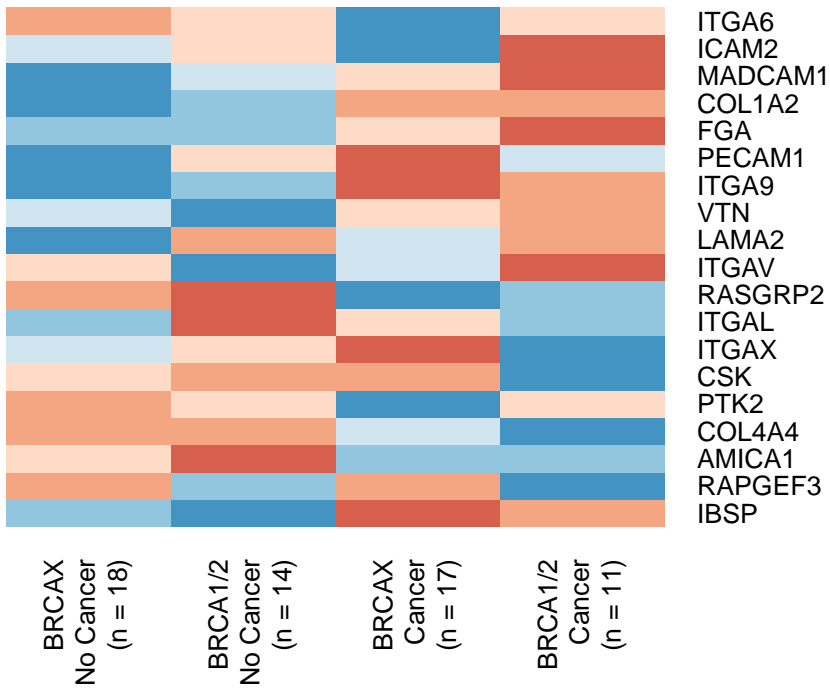

Supplement: Supplementary file 20 — Computer Code EV2 [file MSB-12-860-s020.zip › MSB-15-6506R_Computer_Code_EV2/BCRiskPathways_Notebook/Figures/Figure_S4_Integrin_Ontario.pdf]

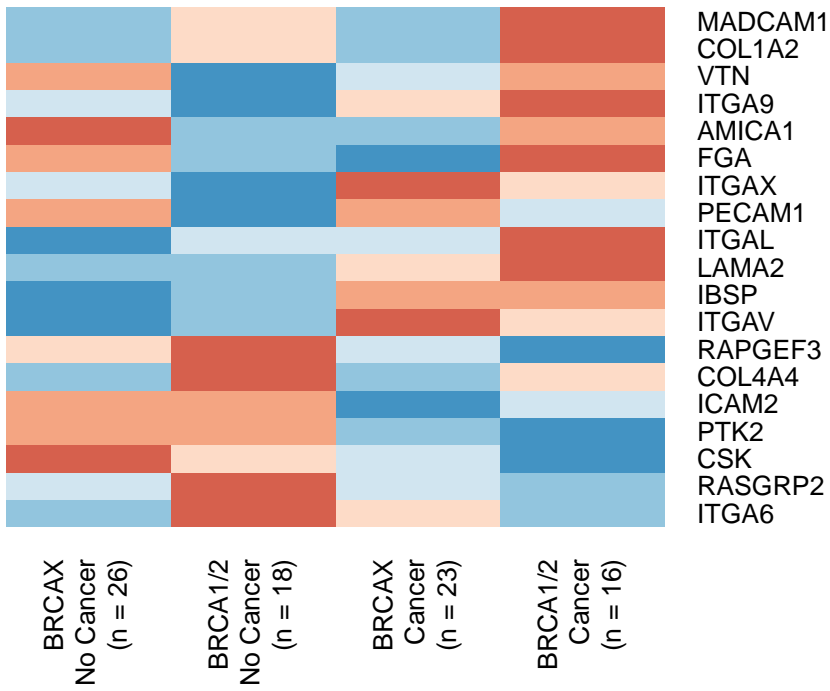

Supplement: Supplementary file 20 — Computer Code EV2 [file MSB-12-860-s020.zip › MSB-15-6506R_Computer_Code_EV2/BCRiskPathways_Notebook/Figures/Figure_S4_Integrin_Utah.pdf]

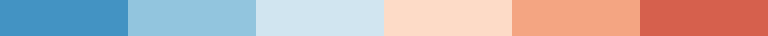

Low

High

Supplement: Supplementary file 20 — Computer Code EV2 [file MSB-12-860-s020.zip › MSB-15-6506R_Computer_Code_EV2/BCRiskPathways_Notebook/Figures/Figure_S4_Legend.pdf]

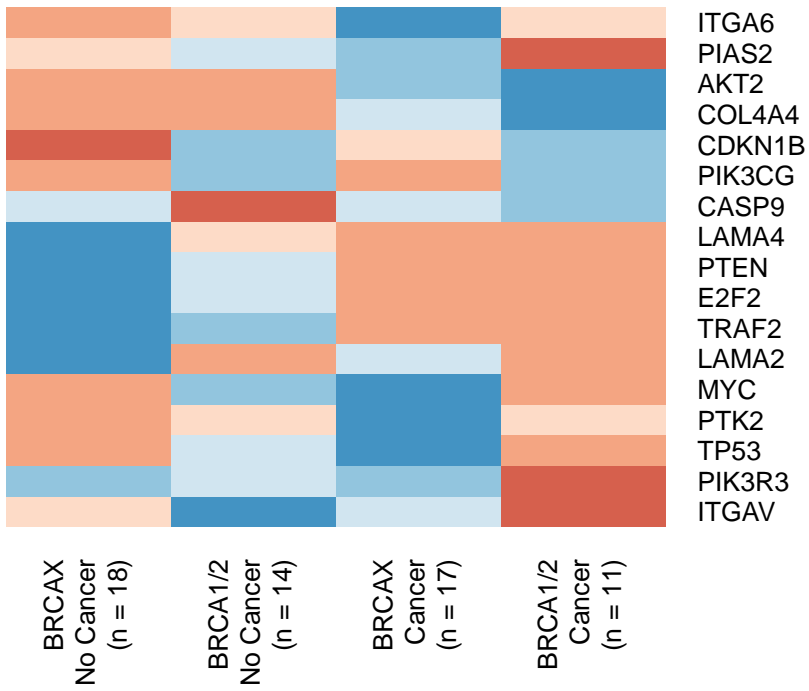

Supplement: Supplementary file 20 — Computer Code EV2 [file MSB-12-860-s020.zip › MSB-15-6506R_Computer_Code_EV2/BCRiskPathways_Notebook/Figures/Figure_S4_SCLC_Ontario.pdf]

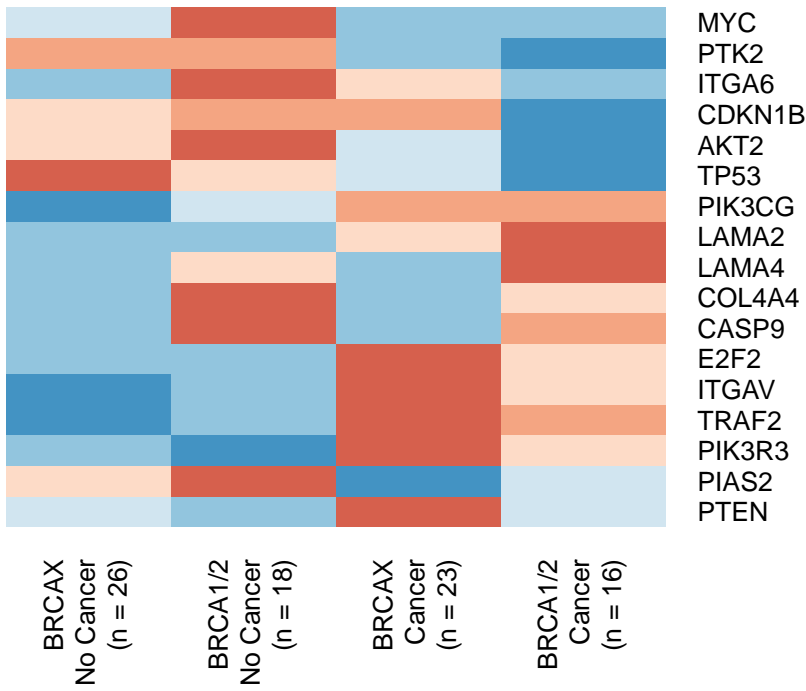

Supplement: Supplementary file 20 — Computer Code EV2 [file MSB-12-860-s020.zip › MSB-15-6506R_Computer_Code_EV2/BCRiskPathways_Notebook/Figures/Figure_S4_SCLC_Utah.pdf]

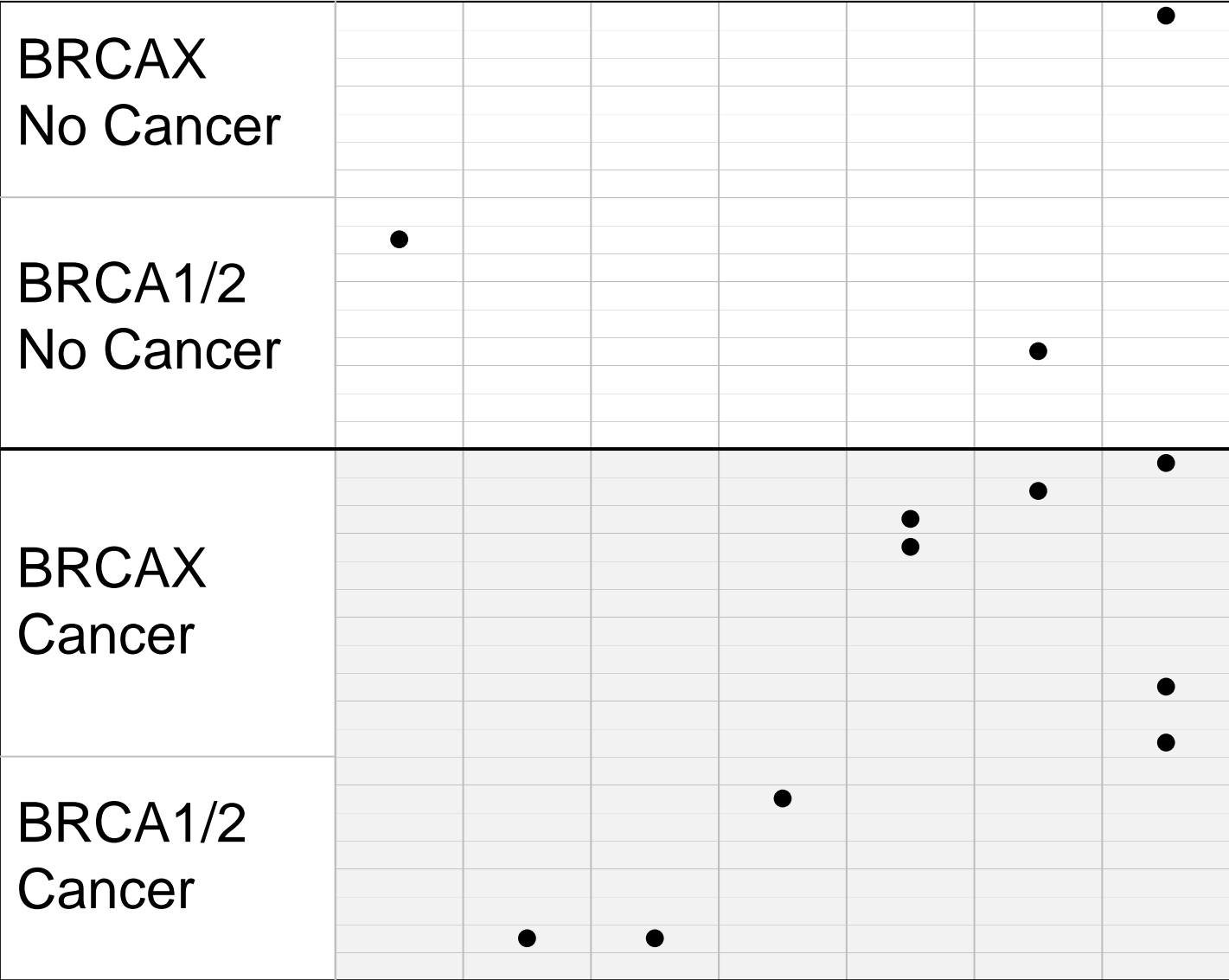

CASP9

CCNE1

ITGA3

NFKB1

PIK3CA

PIK3R5

TRAF2

Supplement: Supplementary file 20 — Computer Code EV2 [file MSB-12-860-s020.zip › MSB-15-6506R_Computer_Code_EV2/BCRiskPathways_Notebook/Figures/Figure_S5_SCLC.pdf]
