# Supplementary material for: Integrative analyses reveal signaling pathways underlying familial breast cancer susceptibility
Source: Mol Syst Biol. 2016 Mar 11;12(3):860. doi: 10.15252/msb.20156506 (PMC4812528; doi:10.15252/msb.20156506)
Supplement: Supplementary file 20 — Computer Code EV2 [file Figure_S5_Integrin.pdf › MSB-15-6506R_Computer_Code_EV2/BCRiskPathways_Notebook/Figures/Figure_S5_Integrin.pdf]

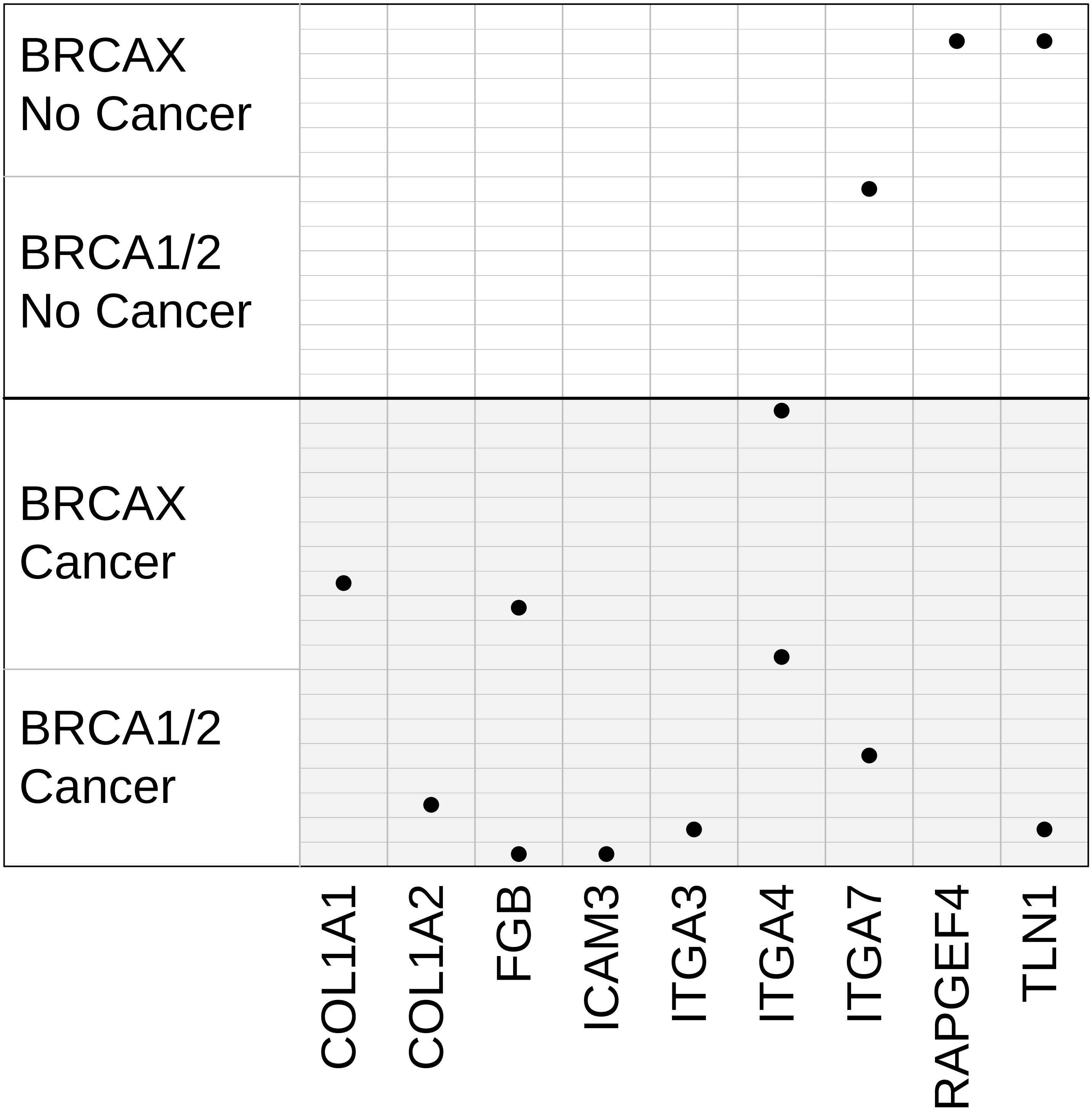
BRCAX
No Cancer
BRCA1/2
No Cancer
BRCAX
Cancer
BRCA1/2
Cancer
COL1A1
COL1A2
FGB
ICAM3
ITGA3
ITGA4
ITGA7
RAPGEF4
TLN1
